# Early cross-coronavirus reactive signatures of humoral immunity against COVID-19

**DOI:** 10.1126/sciimmunol.abj2901

**Published:** 2021-09-09

**Authors:** Paulina Kaplonek, Chuangqi Wang, Yannic Bartsch, Stephanie Fischinger, Matthew J. Gorman, Kathryn Bowman, Jaewon Kang, Diana Dayal, Patrick Martin, Radoslaw P. Nowak, Alexandra-Chloé Villani, Ching-Lin Hsieh, Nicole C. Charland, Anna L.K. Gonye, Irena Gushterova, Hargun K. Khanna, Thomas J. LaSalle, Kendall M. Lavin-Parsons, Brendan M. Lilley, Carl L. Lodenstein, Kasidet Manakongtreecheep, Justin D. Margolin, Brenna N. McKaig, Maricarmen Rojas-Lopez, Brian C. Russo, Nihaarika Sharma, Jessica Tantivit, Molly F. Thomas, Moshe Sade-Feldman, Jared Feldman, Boris Julg, Eric J. Nilles, Elon R. Musk, Anil S. Menon, Eric S. Fischer, Jason S. McLellan, Aaron Schmidt, Marcia B. Goldberg, Michael R. Filbin, Nir Hacohen, Douglas A. Lauffenburger, Galit Alter

**Affiliations:** 1 Ragon Institute of MGH, MIT, and Harvard, Cambridge, MA, USA.; 2 Department of Biological Engineering, Massachusetts Institute of Technology, Cambridge, MA, USA.; 3 Space Exploration Technologies Corporation, Hawthorne, CA, USA.; 4 Department of Cancer Biology, Dana-Farber Cancer Institute, Boston, MA, USA.; 5 Department of Biological Chemistry and Molecular Pharmacology, Harvard Medical School, Boston, MA, USA.; 6 Broad Institute of Massachusetts Institute of Technology (MIT) and Harvard, Cambridge, MA, USA.; 7 Massachusetts General Hospital Cancer Center, Department of Medicine, Massachusetts General Hospital, Boston, MA, USA.; 8 Department of Medicine, Harvard Medical School, Boston, MA, USA.; 9 Center for Immunology and Inflammatory Diseases, Department of Medicine, Massachusetts General Hospital, Boston, MA, USA.; 10 Department of Molecular Biosciences, The University of Texas at Austin, Austin, TX, USA.; 11 Department of Emergency Medicine, Massachusetts General Hospital, Boston, MA, USA.; 12 Department of Microbiology, Harvard Medical School, Boston, MA, USA.; 13 Division of Infectious Diseases, Department of Medicine, Massachusetts General Hospital, Boston, MA, USA.; 14 Division of Gastroenterology, Department of Medicine, Massachusetts General Hospital, Boston, MA, USA.; 15 Brigham and Women’s Hospital, Boston, MA, USA.; 16 Department of Immunology and Infectious Diseases, Harvard TH Chan School of Public Health, Boston, MA, USA.

## Abstract

The introduction of vaccines has inspired hope in the battle against SARS-CoV-2. However, the emergence of viral variants, in the absence of potent antivirals, has left the world struggling with the uncertain nature of this disease. Antibodies currently represent the strongest correlate of immunity against SARS-CoV-2, thus we profiled the earliest humoral signatures in a large cohort of acutely ill (survivors and nonsurvivors) and mild or asymptomatic individuals with COVID-19. Although a SARS-CoV-2–specific immune response evolved rapidly in survivors of COVID-19, nonsurvivors exhibited blunted and delayed humoral immune evolution, particularly with respect to S2-specific antibodies. Given the conservation of S2 across β-coronaviruses, we found that the early development of SARS-CoV-2–specific immunity occurred in tandem with preexisting common β-coronavirus OC43 humoral immunity in survivors, which was also selectively expanded in individuals that develop a paucisymptomatic infection. These data point to the importance of cross-coronavirus immunity as a correlate of protection against COVID-19.

## Common coronavirus antibodies correlate to COVID-19 severity

There are many correlates of protection to SARS-CoV-2 infection, but it is unclear how previously established immune responses to common seasonal coronaviruses might correlate to COVID-19 protection. Here, Kaplonek and Wang *et al.* looked at the early antibody responses to SARS-CoV-2 and the common cold β-coronavirus OC43 in the blood of COVID-19 patients with asymptomatic, moderate, severe, or deadly disease. They found that initial antibody responses to SARS-CoV-2 correlated with antibodies against OC43 in patients who survived COVID-19 and experienced milder disease. Thus, this work suggests that immunity to common seasonal coronaviruses may help to protect against SARS-CoV-2 infection.

## Teaser

Preexisting cross-coronavirus immunity influences the early development of SARS-CoV-2 humoral immunity after COVID-19.

## INTRODUCTION

The relentless spread and unpredictable nature of disease caused by severe acute respiratory syndrome coronavirus 2 (SARS-CoV-2) continues to paralyze the globe. However, the introduction of potent vaccines has inspired new hope that the end of the pandemic is in sight (*1*–*3*). Unfortunately, the slow vaccine rollout, emergence of new viral variants (*4*–*6*), confusing results of convalescent plasma trials, and incomplete efficacy from monoclonal therapeutics, coupled with the lack of potent antiviral therapeutics, have left the globe with a burden of managing the uncertain nature of this disease. Thus, there is an urgent and continued need to characterize the humoral antibody response to acute disease and its correlate with outcomes, to better define biomarkers to support clinical care, and to target the design of monoclonal antibody therapeutics strategies.

SARS-CoV-2–infected patients experience a wide range of clinical manifestations ranging from asymptomatic infection to severe disease that may exacerbate and result in acute respiratory distress syndrome and ultimately death (*7*). However, although age (*8*) and comorbidities have been linked to more severe disease (*9*–*12*), the outcome of SARS-CoV-2 infection is unpredictable. Emerging immune correlate analyses have suggested that early robust neutralizing antibody responses (*13*–*15*), innate immune responses (*16*), Fc receptor (FcR) activity (*17*, *18*), and altered B cell and T cell frequencies (*19*, *20*) and viral loads (*21*–*23*) are all linked to differential outcome.

Among these emerging correlates, antibodies are implicated in both natural resolution of infection (*16*, *21*, *24*) and protection after vaccination (*25*, *26*) with antibody-mediated effector functions observed before the evolution of neutralizing antibody activity (*3*, *27*, *28*). Beyond neutralization, antibodies control or clear infection by leveraging the immune system via their constant domain (Fc) functions that evolve promptly after natural infection (*16*). Specifically, antibodies targeting the S2 domain, the most conserved region of the SARS-CoV-2 spike, evolve earliest and predict survival of natural SARS-CoV-2 infection (*16*). Moreover, S2-specific neutralizing antibodies have been observed in plasma samples collected before the pandemic (*29*, *30*). However, whether these responses emerge from preexisting common coronavirus (cCoV) immune responses remains unclear.

Thus, using samples from a large acute SARS-CoV-2 infection study (*31*), we profiled the evolving humoral immune response to SARS-CoV-2 over the first 12 days after symptom onset and in a community-based mild infection cohort. Class-switched SARS-CoV-2 S2-specific humoral immune responses evolved rapidly and selectively in acute survivors of COVID-19. Moreover, immunoglobulin M (IgM) and IgG responses to the common β-CoV OC43 were expanded in individuals that survived infection and experienced milder disease. Furthermore, the earliest OC43-specific responses were linked to the acutely evolving SARS-CoV-2 responses across the disease spectrum, pointing to the importance of leveraging preexisting cCoV immunity to control and ultimately clear the infection across the disease spectrum. Thus, rather than original antigenic sin, preexisting cross-CoV immunity may accelerate the evolution of cross-reactive humoral immune responses to highly conserved regions of the virus, which may point to critical targets of CoV immunity.

## RESULTS

### Dampened SARS-CoV-2 humoral immune evolution is a signature of COVID-19 mortality

**Fig. 1.**
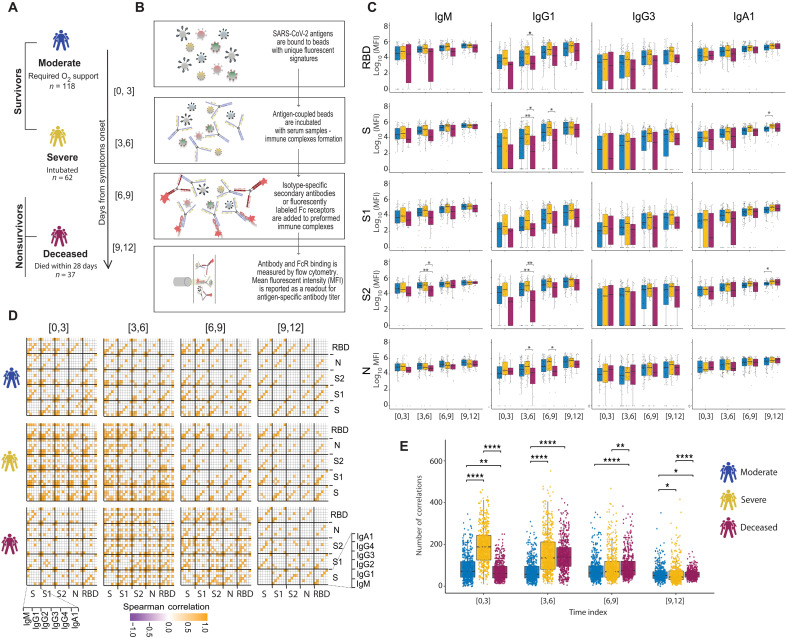
Evolution of early SARS-CoV-2–specific humoral immune responses after symptom onset across acutely ill patients with COVID-19. (**A**) The cartoon shows the study groups on the basis of COVID-19 severity: 217 COVID-19–infected patients were sampled on days 0, 3, and 7 after admission to the hospital. Patients were classified into three groups on the basis of the maximal acuity within 28 days of enrollment. Moderate in blue: Hospitalized that required supplemental oxygen (*n* = 118). Severe in yellow: Intubation, mechanical ventilation, and survival to 28 days (*n* = 62). Deceased in purple: Death within 28 days (*n* = 37). On the basis the day of symptom onset, the samples were divided into four temporal groups: [0, 3], [3, 6], [6, 9], and [9, 12]. (**B**) A graphical summary of the Luminex assay. (**C**) The whisker plots show the distribution of antibody titers across moderate (blue), severe (yellow), and deceased (purple) over the study time course. The solid black line represents the median, and the box boundary (top and bottom) represents the first and third quartiles. The dots show the scaled values of each sample. A two-sample Wilcox test was used to evaluate statistical differences across groups for all the intervals and features. The *P* values were corrected from multiple hypothesis testing using the Benjamini-Hochberg procedure per each interval. Significance corresponds to adjusted *P* values (*P < 0.05 and **P < 0.01). (**D**) The correlation heatmap shows pairwise Spearman correlation matrices of SARS-CoV-2–specific antibody response across COVID-19 severity groups (moderate, severe, and deceased) for all four intervals. Correlation coefficients are shown only if they are larger than 0.6 and statistically significant after Benjamini-Hochberg correction for multiple hypothesis testing. Negative correlations are indicated in purple, and positive correlations are shown in orange. (**E**) The statistical evaluation of the effect of sample size. The Spearman correlation is calculated by randomly selected 10 samples per category for 500 runs. The number of statistically significant correlations (larger than 0.6) is calculated and tested by the Mann-Whitney *U* test. Significance corresponds to adjusted *P* values (*P < 0.05, **P < 0.01, and ****P < 0.0001). RBD, receptor binding domain; S, spike; S1 and S2, subunit 1 and 2 of the spike protein; N, nucleocapsid.

Previous studies have noted distinct humoral evolutionary trajectories across individuals with different clinical outcomes after SARS-CoV-2 infection, marked by different magnitudes of humoral immune responses (*16*, *32*), differential targeting of antigens (*21*), increased functional breadth (*32*), incomplete IgG class switching (*33*), or neutralization activity (*14*). However, many of these studies probed the humoral immune response several days to weeks after symptom onset. Thus, to gain insights into the earliest host-pathogen interactions that may underline differences in disease trajectory, we profiled a cohort of acutely ill patients with COVID-19. A total of 217 patients with confirmed SARS-CoV-2 infection by nasopharyngeal polymerase chain reaction (PCR) were collected at the time of admission through the Emergency Department (ED) at about 0 to 12 days after symptom onset and stratified by disease severity and 28-day outcome into three groups: (i) moderate, requiring hospitalization and supplemental oxygen support (*n* = 118, corresponding to a max score of 4 on the World Health Organization (WHO) Ordinal Outcomes scale in 28 days); (ii) severe, intubated survivors up to 28 days (*n* = 62, corresponding to max WHO scales of 6 to 7); and (iii) deceased, nonsurvivor (*n* = 37, corresponding to WHO scale 8) (Fig. 1A and table S1) (*31*, *34*).

System Serological profiling (Fig. 1B) during the acute window of infection pointed to a significant deficit in IgG1 receptor binding domain (RBD), full S protein, S2, N-specific antibody, and S2-specific IgM levels in the nonsurvivor group between 3 and 9 days after symptom onset (Fig. 1C). Similar trends were noted for SARS-CoV-2–specific IgG3 and IgA1 titers and Fcγ receptor (FcγR) binding capacity (fig. S1). Specifically, SARS-CoV-2–specific antibodies able to bind to low affinity FcRs (FcγR2A, FcγR2B, FcγR3A, and FcγR3B) were generated at lower levels in nonsurvivors compared with COVID-19 survivors (fig. S1). Moreover, although FcγR binding was associated with neutralization in survivors of COVID-19, this relationship was lost in individuals that ultimately passed away (fig. S2). Overall, humoral immune responses were poorly coordinated in the nonsurvivors compared with individuals that experienced moderate or severe disease.

Given that antibodies are generated as polyclonal swarms, we next profiled the coordination of the evolution of the humoral immune response across the groups. Higher coordination was observed in severe disease survivors within 0 to 3 days of symptom onset compared with the limited coordination observed in the moderate and nonsurvivor groups (Fig. 1D), with higher overall levels of coordination in the severe survivors (Fig. 1E). Conversely, coordination in the humoral immune response increased at days 3 to 6 and immediately decreased in individuals that did not survive SARS-CoV-2 infection but persisted in individuals with moderate disease. This analysis suggests that severe survivors of COVID-19 generate a more robust, highly coordinated acute humoral immune responses very early in disease compared with patients who succumbed to COVID-19.

### S2-specific responses are selectively enriched in survivors of COVID-19

**Fig. 2.**
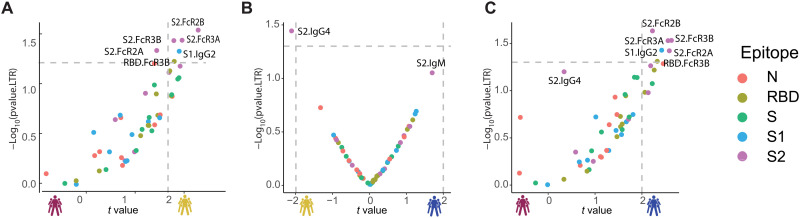
Selective enrichment of S2-specific responses across patients with COVID-19. (**A** to **C**) Volcano plots of pairwise comparisons across pairs of each of the three groups highlight differences across groups controlling for age, body mass index, heart, lung, and kidney diseases. The volcano plots include comparisons of (A) individuals that passed away within 28 days (deceased) versus severe survivors, (B) individuals who experienced moderate disease versus severe survivors, and (C) individuals who ultimately passed away (deceased) versus individuals who developed moderate disease. The *x* axis represents the *t* value of the full model, and the *y* axis denotes the *P* values by likelihood ratio test comparing the null model and full model. The null/full model represents the association between each individual measurement (response) and all collected clinical information with/without disease severity (see Methods). The horizontal gray dashed line denotes the *P* value equals 0.05, and the vertical gray dashed line denotes a manually selected threshold (*t* values = 2).

Considering the multitude of differences across the groups, we next aimed to define whether specific longitudinal humoral features could resolve survivors from nonsurvivors. Thus, we generated pairs of nested mixed linear models across each set of subjects, accounting for comorbidities, age, and sex, all attributes previously linked to more severe COVID-19 (Fig. 2, A to C) (*35*). First, we compared the SARS-CoV-2 response across survivors with severe disease to individuals that did not survive severe disease. After correction for multiple comparisons, six features—including S2 FcγR2, S2 FcγR2B, S2 FcγR3A, S2 FcγR3B, S1 IgG2, and RBD FcγR3B—were selectively enriched among patients who survived COVID-19. No features were selectively enriched in nonsurvivors. The four features directed at the highly conserved S2 domain of the SARS-CoV-2 spike included FcR binding activity, rather than antibody IgG titers (Fig. 2A), pointing to an early enrichment of functional antibodies largely directed at the S2 domain as a marker of survival.

In contrast, a more balanced distribution of antibody features was observed when comparing moderate and severe survivors with solely S2-specific IgG4 levels enriched among severe survivors (Fig. 2B), again illustrating the critical value of S2-specific immunity as a marker of disease outcome. Last, the comparison of survivors with moderate infection compared with nonsurvivors highlighted the presence of elevated immune responses in individuals with moderate disease, marked by five statistically significant S2-specific FcR binding antibody features that were selectively enriched among individuals with moderate disease compared with nonsurvivors (Fig. 2C). Collectively, these data highlight the enrichment of S2-specific Fc profiles as early biomarkers of disease outcome, suggesting that these earliest responses may represent either biomarkers of mechanistic responses in the early control or clearance of infection required for survival of COVID-19.

### cCoV responses are enriched in survivors early in infection course

**Fig. 3.**
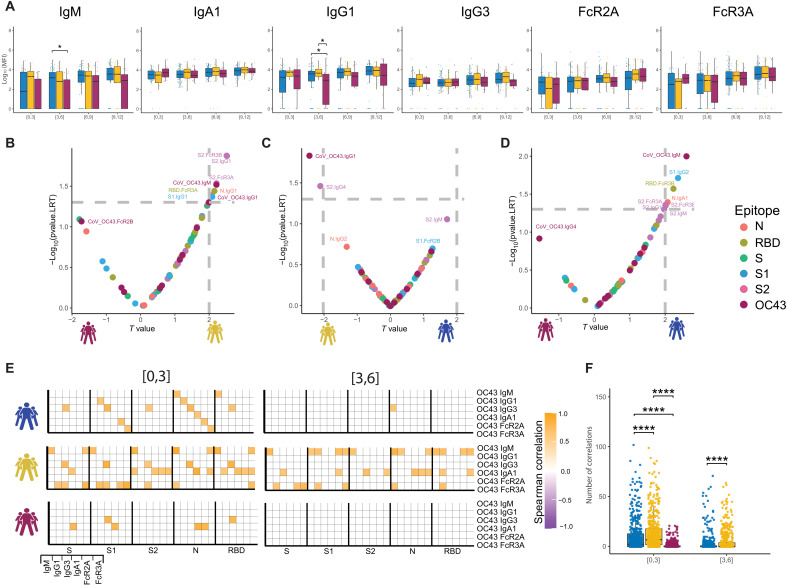
The temporal evolution of the human OC43-specific humoral immune response. (**A**) The whisker bar graphs show the distribution of human OC43 RBD–specific antibody titers and OC43-specific antibody mediated FcR binding profiles across moderate, severe, and nonsurvivor COVID-19 groups over the study time course. The solid black line represents the median and box boundary (top and bottom). (**B** to **D**) The volcano plots show the pairwise comparisons across the three COVID-19 severity groups: (B) individuals that passed away within 28 days (deceased) versus severe survivors, (C) individuals who experienced moderate disease versus severe survivors, and (D) individuals who ultimately passed away (deceased) versus individuals who developed moderate disease, including human OC43 RBD–specific humoral immune data. (**E**) The correlation heatmap shows the pairwise Spearman correlation matrices between OC43-specific and SARS-CoV-2 antibody levels across three COVID-19 severity groups (moderate, severe, and nonsurvivors) across the study time course. The correlation coefficients were shown only if statistically significant (adjusted *P* value < 0.05) after Benjamini-Hochberg correction from multiple hypothesis testing. (**F**) The statistical evaluation of the effect of sample size. The Spearman correlation is calculated by randomly selected 10 samples per category for 500 runs (the deceased group in day interval [3,6] is not included because the number of samples is less than 10). The number of statistically significant correlations (larger than 0.6) is calculated and tested by the Mann-Whitney *U* test. Significance corresponds to adjusted *P* values (*P < 0.05, and ****P < 0.0001).

The near simultaneous evolution of S2-specific IgG and IgM at early time points in survivors (fig. S3), displayed as a ratio of IgM/IgG levels (*13*, *22*, *36*–*38*), suggests either a remarkably rapid maturation of the humoral immune response or the potential expansion of preexisting cross-CoV immunity to the conserved S2 domain. The elevated IgM/IgG ratio observed in nonsurvivors points to a delay in class switching from IgM to IgG in this population (fig. S3). Annual circulation of cCoVs gives rise to broad cross-CoV immunity. However, how this cCoV-specific immunity influences the disease trajectory of COVID-19 remains unclear (*29*, *39*–*45*). Among the cCoV, the β-CoV OC43 circulates annually in the United States (*46*). Thus, to begin to define the relationship between cCoV and SARS-CoV-2 outcomes, we profiled the OC43-specific humoral response across the groups. To avoid the detection of cross-reaction antibodies across OC43 and SARS-CoV-2, immune profiling focused on OC43 RBD antibodies, due to the limited conservation of RBD domains across the β-CoVs. Unexpectedly, higher OC43 RBD–specific IgM and IgG1 levels were observed in survivors with severe and moderate disease, particularly within 3 to 6 days of symptom onset compared with nonsurvivors (Fig. 3A). Conversely, no differences were noted in IgA, IgG3, or FcR binding across the groups. Only a slight increase was observed in all OC43 responses across the groups over the study period, indicating stability in cCoV immunity that was not boosted by infection. These data point to the early enrichment of OC43 immunity, but not an evolution of these responses, in COVID-19 survivors in the first week of observation.

To further define whether the presence of these differential OC43-specific immune responses could resolve the COVID-19 severity groups, we next integrated OC43 RBD–specific humoral immune profiles into the paired nested mixed linear models. Although S2-specific humoral immune responses remained top predictors in severe survivors compared with nonsurvivors, OC43 RBD–specific IgM antibody levels were significantly and selectively also expanded in the severe survivors (Fig. 3B), pointing to the importance of early expanded OC43-IgM levels in resolving disease trajectory alongside the previously observed S2-specific Fc-binding signatures. Likewise, OC43 RBD–specific IgG1 was enriched in survivors with severe disease compared with moderate disease (Fig. 3C), pointing to the potential importance of more mature expanded OC43-response in the resolution of severe rather than moderate disease. Last, OC43 RBD–IgM levels were the most discriminatory feature between individuals with moderate disease and individuals who died within 28 days (Fig. 3D), pointing again to the importance of leveraging preexisting cCoV IgM immunity as a marker of early protective immunity against this respiratory pathogen (*47*, *48*). Thus, these data suggest that the level of preexisting cCoV immunity contributes early in disease to differentiating disease trajectory, with cCoV-IgM levels representing a determinant of survival.

To ultimately determine whether the magnitude of preexisting cCoV immunity could be linked to the evolution of early SARS-CoV-2 immunity across groups, we next examined at the relationship between the earliest OC43 and SARS-CoV-2 responses across all groups over time. Negative correlations would indicate that preexisting cCoV immune responses dampened or blocked the evolution of SARS-CoV-2 immunity, and positive correlations would suggest that the preexisting cCoV-immune response may give rise to evolving SARS-CoV-2 immunity. Notable differences were observed in the overall correlational structure of the earliest OC43 and SARS-CoV-2 responses (Fig. 3, E and F). No negative correlations were observed. Instead, enhanced positive correlations were observed across OC43/SARS-CoV-2 in survivors compared with nonsurvivors, marked by more correlations in survivors with moderate disease, followed by severe disease compared with nonsurvivors, pointing to the importance of rapidly evolving cross-reactive immunity as a marker of enhanced disease control. Relationships were observed across S, S1, S2, nucleocapsid, and RBD, suggesting that preexisting OC43 responses likely mark a more generally cross-reactive immune response, enabling broad SARS-CoV-2–specific humoral immune evolution across antigens. Conversely, these relationships decayed across all groups just 3 days later, possibly suggesting that this relationship was only observable early in infection, before the evolution and divergence of the rapidly evolving SARS-CoV-2–specific affinity matured immune response.

### Expanded S2-specific FcR binding antibodies are selectively enriched in asymptomatic infection

**Fig. 4.**
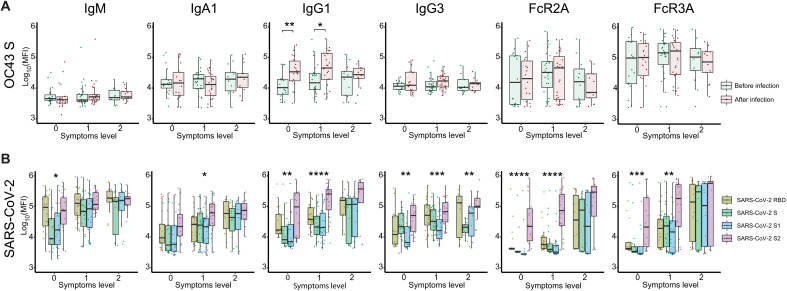
SARS-CoV-2 S2–specific antibody functionality tracks with asymptomatic SARS-CoV-2 infection. (**A**) The whisker box plots show the overall humoral immune response to OC43 RBD–spike titers across a community-based SARS-CoV-2 infection cohort divided by individuals that were asymptomatic (symptoms level 0) or experienced symptoms (symptoms level 1 or level 2, based on degree of symptoms) before and after infection. (**B**) The bar graphs illustrate the SARS-CoV-2–specific humoral immune response across the RBD, S, S1, and S2 antigens across the same community-based surveillance study divided by the degree of symptoms (symptoms levels). The dots show the scaled values of each sample. A two-sample Wilcox test was used to evaluate statistical differences across different epitopes for all the symptom categories. Significance corresponds to adjusted *P* values (*P < 0.05, **P < 0.01, ***P < 0.001, and ****P < 0.0001).

To test whether cCoV cross-reactive immunity was solely a marker of enhanced disease control in hospitalized patients or may also represent a marker of generally milder disease, we extended the analysis of cCoV immunity to a community-based cohort study of asymptomatic/paucisymptomatic SARS-CoV-2 infection. Individuals were sampled both before and after SARS-CoV-2 infection (*49*). Although all individuals harbored robust class-switched IgA and IgG OC43 responses before infection, no differences were observed in OC43-specific antibody profile across asymptomatic individuals (level 0), individuals that evolved a single symptom (level 1), or individuals that had few mild symptoms (level 2) (fig. S4) before or after infection (Fig. 4A). Conversely, after infection, OC43-specific IgG1 were selectively increased in individuals that remained asymptomatic or a single symptom. These data argue that class-switched OC43-specific immunity may also be expanded and linked to more robust containment of asymptomatic/paucisymptomatic SARS-CoV-2 disease. Similarly, robust SARS-CoV-2-specific humoral immune responses (IgM, IgA, IgG1, and IgG3) were noted in both asymptomatic and paucisymptomatic (symptoms level 1 and 2) individuals. However, an unexpected difference was noted in the FcR binding ability of SARS-CoV-2–specific antibodies and specifically in S2-specific humoral immune responses across symptom levels. Specifically, although asymptomatic and individuals that only experienced a single symptom did not elicit S1- or RBD-specific antibodies able to bind to FcRs, these unique individuals solely generated FcR binding S2-specific antibodies (Fig. 4B). At a more granular level, although S2-specific IgG1 humoral immune responses were expanded across individuals, at all symptoms levels, S2-specific antibodies with the capacity to bind to the phagocytic FcγR2A and cytotoxic FcγR3A receptors were elicited in asymptomatic individuals. Conversely, all SARS-CoV-2–specific antibodies had the capacity to interact with FcRs in individuals with more symptoms after mild SARS-CoV-2 infection. Together, these data suggest that the expansion of S2-specific responses may emerge rapidly after infection, derived from preexisting cCoV immunity, and contribute to disease severity via Fc-mediated antiviral control and clearance.

## DISCUSSION

Since its identification in late 2019, SARS-CoV-2 has caused hundreds of millions of infections, more than 4 million deaths, overwhelmed health systems, and affected global economies (*50*, *51*). Public health measures—including masks, distancing, and quarantines—have helped to slow the spread of the virus (*52*, *53*), but the rapid evolution of variants of concern, coupled to the relaxation of public health measures, has led to global increases in spread. Although SARS-CoV-2–specific therapeutics have shown more moderate promise (*54*), vaccines are likely to be the key to ending the pandemic. However, with the rise of SARS-CoV-2 variants that evade vaccine-mediated neutralization, the development of next-generation vaccines or boosting strategies has been difficult in the absence of precise correlates of immunity against COVID-19.

However, beyond neutralization, Fc effector functions have been linked to the resolution of natural infection (*55*–*58*) and vaccine-induced protection (*4*, *59*, *60*). Moreover, Fc effector functions have also been mechanistically implicated in the efficacy of particular monoclonal therapeutics against SARS-CoV-2 (*61*–*64*). Moreover, early spike-specific Fc effector functions, mainly targeting the S2, were previously observed among survivors (*16*, *21*) and prepandemic S2-specific neutralizing antibodies have been observed primarily in children (*29*) who are typically spared from COVID-19 (*65*). However, the precise origin and specificity of these early and/or preexisting S2-reactive antibodies were unclear. Here, using acute hospitalized patients with COVID-19, an association was observed between FcR binding S2-specific humoral immune responses and survival COVID-19. Specifically, S2 class-switched IgG antibodies, with the capability of binding to multiple FcRs, were observed just days after symptom onset, pointing to the rapid emergence of SARS-CoV-2 functional humoral immunity, likely from some preexisting humoral reservoir, rather than solely attributable to de novo evolution of these responses. The observation concurs with previous data, substantiating the earliest evolution of S2-specific immunity, with other specificities evolving more slowly, in an orthogonal cohort (*16*). Given the conserved nature of S2 across β-CoVs, these data suggested that individuals who survive COVID-19 may have an earlier advantage as they may be able to rapidly redeploy S2-specific antibodies across CoVs to combat disease.

Unlike the potent neutralizing activity of antibodies against RBD, S2-specific antibodies are more weakly neutralizing (*66*–*68*) and thus likely provide protection through additional humoral mechanisms (*16*). Like the influenza stem-specific antibodies that contribute to protection via Fc-dependent antibody-dependent cellular cytotoxicity (*69*), recent work in animals highlights the importance of Fc-dependent mechanisms for S2-specific monoclonal antibodies (*67*). In our cohort of patients with acute COVID-19, S2-specific antibodies with FcR binding capabilities, rather than S2-titers alone, were among the strongest correlates of protective immunity against death. Moreover, S2-specific antibody functions were selectively augmented in asymptomatic SARS-CoV-2 infection, suggesting that the ability of these antibodies to recruit specific innate immune effector functions may be key to their protective activity. Thus, even in the absence of potent neutralization, S2-specific antibody effector functions may be key to the early recognition, control, and clearance of viruses resulting in attenuated disease. Thus, unlike RBD-specific antibodies that may be critical for driving sterilizing immunity, S2-specific antibodies may be key to attenuating disease (*67*). Moreover, given the highly conserved nature of S2, it is plausible that highly functional S2-specific monoclonal antibodies or vaccine-induced immune responses may provide broader and more potent protection against emerging SARS-CoV-2 variants and perhaps even additional β-CoVs. Thus, despite the increasing numbers of infections observed globally in the wake of the emergence of the highly contagious Delta (B.1.617.2) variant, rates of hospitalization have not increased proportionally (*70*). Whether this protection is related to the ability of vaccines to drive robust S2-specific immune responses remains unclear but could account for high levels of breakthroughs in the absence of disease.

S2-specific responses exhibit delayed kinetics in nonsurvivors compared with severe and moderate survivors of COVID-19. Thus, although current RBD-specific monoclonal therapeutics have struggled to compete with preexisting S1/RBD-specific antibodies that were already present in the circulation of severely ill individuals (*71*), it is plausible that the administration of highly functional antibodies to a less immunodominant target in vulnerable populations could represent a critical opportunity to improve disease outcomes. Thus, the delivery of S2-specific monoclonal antibodies may offer an opportunity to supplement a missing population of antibodies to support the response to SARS-CoV-2 across a broader clinical window of therapeutic opportunity to control and clear the infection. Hence, identification of the most potent S2 antibodies, linked to key antibody effector function, may shift the host-pathogen interaction and drive enhanced protective immunity.

The presence of higher acute OC43-specific humoral immune response among patients with better clinical outcomes raised the possibility that cross-reactive SARS-CoV-2–specific immunity may emerge from preexisting OC43-specific humoral memory. Unexpectedly, OC43-specific IgG and IgM antibodies were among the top discriminatory features in survivors. Specifically, OC43-specific IgG was an acute marker of survival of severe disease, whereas OC43-specific IgM response was a marker of moderate disease. Although traditionally, IgM is considered a marker of a newly emerging immune response, mounting data suggest that IgM responses can persist throughout infection, continue to affinity mature, and remerge to fight infection from memory (*47*, *48*, *72*). For example, some of the most potent universal influenza-specific antibodies have been cloned from affinity-matured IgM^+^ memory B cells (*72*, *73*), pointing to the importance of IgM memory as a critical source of potentially protective antibodies in case of respiratory infections. Given that the remarkable avidity and functional potency of IgM able to drive robust complement and opsonophagocytic activity (*74*–*76*), it is plausible that IgM responses may be sufficient in the context of acute infection to drive rapid control and clearance of the pathogen. Moreover, SARS-CoV-2–specific IgM monoclonal therapeutics are more potent than IgG therapeutics in the treatment of SARS-CoV-2 infection in animal models (*77*–*79*), potentially pointing to the possibility that individuals able to selectively expand their OC43-specific IgM or IgG will respond better to COVID-19.

We did not see any interference or evidence of antigenic imprint shaped by the presence of preexisting cross-CoV immunity. Thus, unlike original antigenic sin in the context of influenza where preexisting immunity to a particular strain of influenza prevents boosting to orthogonal contemporaneous strains (*80*), we did not observe any evidence of anticorrelated SARS-CoV-2 immunity depending on the magnitude or quality of the preexisting OC43-specific cCoV immune response. We observed global coordination of SARS-CoV-2–specific humoral immune responses with OC43 in survivors, albeit these relationships did not exist or were subdued in nonsurvivors. Because the RBD and S1 share limited homology across the viruses, it is less likely that preexisting responses to OC43 could directly lead to the evolution of S1- or RBD-specific responses. However, the presence of class-switched OC43-specific humoral immunity is likely accompanied by a robust cCoV–T cell immune response across the viral proteome that is likely to also play a key part in the accelerated evolution of the humoral immune response across additional conserved antigenic targets including S2 and the nucleocapsid. Thus, whether S2 antibodies alone or preexisting cross-CoV–specific T cells contribute to enhanced control of acute infection remains unclear but points to a potential intersection between the humoral and cellular immune response, to conserved sites, that may be key to protective immunity against SARS-CoV-2.

Collectively, despite our inability to capture individuals before symptom onset, our study provided an opportunity to map humoral immune responses from nearly the time of symptom onset that tracked with significantly different clinical outcomes. Deep humoral profiling pointed to the presence of an acute S2-specific FcR binding signature as a marker of survival of disease and reduced symptomatology, likely evolving from early preexisting robust cCoV humoral immunity. Although these S2-specific humoral immune responses are likely to permit breakthrough infections, the development of future vaccines or boosting regimens that able to promote immunity to this highly conserved domain of SARS-CoV-2 may provide broad protection against emerging variants of concern and even other CoVs.

## METHODS

### Study design

The objective of the study was to define humoral profiles that could predict differences in clinical trajectory after SARS-CoV-2 infection. The study began in March 2020, focused on comparing acutely ill patients with COVID-19 presenting to the emergency room at the Massachusetts General Hospital (MGH) or from a community-based surveillance study run through Space Exploration Technologies Corporation. Acute subjects were categorized by the clinical team. This study was blinded until the final analysis. All experiments were performed in technical replicates. The study protocol was approved by MGH and the Western Institutional Review Board. All participants provided written informed consent.

### Patient cohort and clinical data collection

#### Acutely ill patients with COVID-19

Patients 18 years or older (*n* = 384) with acute respiratory distress and clinical concern for COVID-19 were enrolled in the ED in Boston during the peak of the COVID-19 surge (from 24 March 2020 to 30 April 2020), 306 of whom tested positive for SARS-CoV-2 nasopharyngeal PCR as described by Filbin *et al.* (*31*). This analysis included SARS-CoV-2–positive patients whose symptoms onset was between 0 and 12 days before presentation and whose illness severity and 28-day outcome were classified into three groups; (i) moderate, hospitalized and requiring oxygen support but not mechanical ventilation (*n* = 118, corresponding to WHO Ordinal Outcomes scale 4); (ii) severe, intubated but survived to 28 days (*n* = 62, corresponding to WHO scales 6 to 7); and (iii) deceased within 28 days, nonsurvivor (*n* = 37, corresponding to WHO scale 8) (*34*). Of the 42 patients with COVID-19 who died, 24 (57%) received mechanical ventilation, and 18 (43%) did not. Patients were excluded from analysis if they were discharged directly from the ED and were not hospitalized within the next 28 days or if they were admitted but did not require supplemental oxygen.

Day 0 blood samples were obtained with the initial clinical blood draw in the ED, and day three and day seven samples were obtained during patients’ hospitalization. The clinical course was followed for 28 days after enrollment to establish outcomes. Samples and clinical information were collected according to an Institutional Review Board–approved protocol (*31*). Symptom duration upon presentation was obtained via chart review. Demographic, medical history, and clinical data were collected and summarized for each outcome group using medians with interquartile ranges and proportions with 95% confidence intervals where appropriate.

#### Community-acquired mild and asymptomatic COVID-19 individuals

Industry employees (Space Exploration Technologies Corporation) included volunteers tested for COVID-19 starting in mid-April 2020. All employees were invited to participate by email, and there were no exclusion criteria. Participants completed a study survey including the collection of COVID-19–related symptoms (*49*). Upon obtaining informed consent, blood samples were collected approximately every 39.7 days (SD, 13.8 days). Symptoms were classified by severity, with two points being assigned to loss of smell/taste, fever, feverish/chills, or cough, and one point being assigned to other symptoms such as increased fatigue, headache, congestion, nausea/vomiting, diarrhea, sore throat, and body/muscle aches. Symptom scores were summed, and each subject was categorized into one of three levels based on degree of symptoms: level 0 (*n* = 18), no symptoms; level 1 (*n* = 27), mild (symptom score 1 to 5); and level 2 (*n* = 13), moderate (symptom score 6 to 14).

### Luminex

SARS-CoV-2– and eCoV-specific antibody subclass/isotype and FcγR binding levels were assessed using a 384-well–based customized multiplexed Luminex assay, as previously described (*81*). SARS-CoV-2 RBD (provided by A. Schmidt, Ragon Institute), SARS-CoV-2 nucleocapsid (N) protein (Aalto BioReagents), and SARS-CoV-2 spike protein (S) (provided by E. Fischer and D. Farber), SARS-CoV-2 subunit 1 and 2 of the spike protein (S1 and S2) (Sino Biological), and as human eCoV antigens: hCoV-OC43 RBD (provided by A. Schmidt, Ragon Institute), hCoV-OC43 spike protein (S) (Sino Biological), hCoV-HKU1 spike protein (S) (ImmuneTech), SARS-CoV-1, Middle East respiratory syndrome spike proteins (S) (provided by J. McLellan, University of Texas) were used to profile specific humoral immune responses. A mix of hemagglutinin (HA) A/Michigan/45/2015 (H1N1), HA A/Singapore/INFIMH-16-0019/2016 (H3N2), and B/Phuket/3073/2013 (ImmuneTech) was used as a control. Antigens were coupled to magnetic Luminex beads (Luminex Corporation) by carbodiimide–*N*-hydroxysuccinimide ester coupling (Thermo Fisher Scientific). Antigen-coupled microspheres were washed and incubated with plasma samples at an appropriate sample dilution (1:500 for IgG1 and all FcγRs and 1:100 for all other readouts) for 2 hours at 37°C in 384-well plates (Greiner Bio-One). Unbound antibodies were washed away, antigen-bound antibodies were detected by using a phycoerythrin-coupled detection antibody for each subclass and isotype (IgG1, IgG2, IgG3, IgG4, IgA1, and IgM; Southern Biotech), and FcγRs were fluorescently labeled with PE before addition to immune complexes (FcγR2A, FcγR2B, FcγR3A, and FcγR3B; Duke Protein Production facility). After 1-hour incubation, plates were washed, and flow cytometry was performed with an IQue (Intellicyt), and analysis was performed on IntelliCyt ForeCyt (v8.1). PE median fluorescent intensity (MFI) is reported as a readout for antigen-specific antibody titers.

### Quantification and statistical analysis

All analyses were performed using R version 4.0.3. All the figures were created with many R-supported packages, mainly including ggplot, ggrepel, and ggpubr.

#### Data preprocessing

The raw MFI from flow cytometry was scaled by the log_10_ function. To control for noise, phosphate-buffered saline values were subtracted from the scaled titer measurements.

#### Univariate plots

The box-plots summarize the median value with first and third quantiles for each clinical group (moderate, severe, and deceased) across day ranges from symptom onset within an interval of 3 days (day ranges 0 to 3, 3 to 6, 6 to 9, and 9 to 12). Paired *P* value were defined by the Mann-Whitney *U* test for each subfeature on the individual temporal course across different clinical groups and adjusted by the Benjamini-Hochberg procedure of multiple testing correction. The visualization was performed by the function “ggplot” of R package “ggplot2” (3.3.3), and the *P* value was estimated by the function “wilcox_test” and “adjust_pvalue” in the R package “rstatix” (0.6.0) and labeled by the function “stat_pvalue_manual” in the R packge “ggpubr” (0.4.0).

#### Correlation analysis

Spearman correlations were used to evaluate the relationship between different measurements and were performed using the function “rcorr” of R package “Hmisc” (4.4.2). A time-specific correlation analysis between different antibody measurements was used to explore temporal coordination between features. Specifically, Spearman coefficients were defined between titer values across time points within each clinical group. The significance of correlation was adjusted by the Benjamini-Hochberg procedure of multiple testing correction.

To avoid the impact of differing sample sizes, we evaluated the effect of different sample sizes across disease severities and time intervals, two strategies were applied: (i) First, for the purpose of visualization, correlation coefficients were considered only if larger than 0.6, given that coefficients may reach significance more easily using larger sample sizes; (ii) a down-sampling strategy was also explored to equalize the sample size across different groups for statistical evaluation. For the down-sampling, we randomly down-sampled 10 samples and calculated the Spearman correlation for 500 random sampling runs. Then, the number of significant correlations larger than 0.6 were calculated and tested by the Mann-Whitney *U* test, and multiple testing correction was implemented to avoid statistical anomalies.

#### Defining signatures of disease outcome while also controlling for potential cofounders

We accessed the significance of the association between measured antibody levels and clinical outcomes by controlling for collected potential cofounders using two nested mixed linear models (null and full model) without/with clinical outcomes. We fit two linear mixed models and estimated the improvement in model fit by likelihood ratio testing to assess how many measurements have a significantly better fit with the full model at a threshold of <0.05.

Null model

$$\begin{array}{c} \text{antibodymeasurement}\sim \text{TimeFactor}+\\ \text{well}.\text{plate}+\text{physical}.\text{information}+\\ \text{historical}.\text{diseases}+\text{TimeFactor}*\\ \text{Symptom}+\text{TimeFactor}*\\ \text{immunemeasurement}+(1|\text{Pat}) \end{array}$$

Full model

$$\begin{array}{c} \text{antibodymeasurement}\sim \text{TimeFactor}+\\ \text{well}.\text{plate}+\text{physical}.\text{information}+\\ \text{Clinical}.\text{outcome}+\text{historical}.\text{disease}+\\ \text{TimeFactor}*\text{Symptom}+\text{TimeFactor}*\\ \text{immune}.\text{measurement}+(1|\text{Pat}) \end{array}$$

Likelihood ratio test $\mathrm{LRT}=-2*\text{ln}(\frac{\text{MLE in Full model}}{\text{MLT in Null model}})\sim \lambda^{2}$

Here, the historical.diseases include comorbidities such as heart, lung, kidney, and diabetes, whereas physical.information included age and body mass index. Well.plate represented the batch indicator on the Luminex platform. Time-related symptoms included respiratory symptom, fever, gastrointestinal symptoms, and other immune measurements, such as absolute neutrophil count, absolute monocyte count, creatinine, the level of C-reactive protein, d-dimer, lactate dehydrogenase along the time, and troponin level at 72 hours. The R package “lme4” was used to fit the linear mixed model to each measurement and test for measurement across the contrast of interest. The *P* value from the likelihood ratio test and *t* value of Clinical outcome in full model were visualized as volcano plot using the ggplot function in R package ggplot2.

#### Statistics

Statistical analyses were performed using R ggpubr and ggcorrplot packages. Statistical significance between groups was determined using two-sided Mann-Whitney tests to compare ranks implemented in the *wilcox_test* function of R rstatix package. *P* values of <0.05 were considered significant. In addition, the *P* values were adjusted by Benjamini-Hochberg multiple testing correction.

## Supplementary Material

20210909-1

## Notes

View/request a protocol for this paper from *Bio-protocol*.
