# Supplementary material for: Early cross-coronavirus reactive signatures of humoral immunity against COVID-19
Source: Sci Immunol. 2021 Sep 9;6(64):eabj2901. doi: 10.1126/sciimmunol.abj2901 (PMC8943686; doi:10.1126/sciimmunol.abj2901)
Supplement: 20210909-1 [file sciimmunol.abj2901.v1.pdf]

Cite as: P. Kaplonek *et al.*, *Sci. Immunol.*
10.1126/sciimmunol.abj2901 (2021).

CORONAVIRUS

Early cross-coronavirus reactive signatures of humoral immunity against COVID-19

Paulina Kaplonek[1*], Chuangqi Wang[2*], Yannic Bartsch[1], Stephanie Fischinger[1], Matthew J. Gorman[1], Kathryn Bowman[1], Jaewon Kang[1], Diana Dayal[3], Patrick Martin[3], Radoslaw P. Nowak[4,5], Alexandra-Chloé Villani[7,8,9,10], Ching-Lin Hsieh[11], Nicole C. Charland[6], Anna L.K. Gonye[7,8], Irena Gushterova[7,8], Hargun K. Khanna[6], Thomas J. LaSalle[7,8], Kendall M. Lavin-Parsons[6], Brendan M. Lilley[6], Carl L. Lodenstein[6], Kasidet Manakongtreecheep[7,10], Justin D. Margolin[6], Brenna N. McKaig[6], Maricarmen Rojas-Lopez[9,13,15], Brian C. Russo[9,13,15], Nihaarika Sharma[7,8], Jessica Tantivit[7,10], Molly F. Thomas[7,8,9,10,16], Moshe Sade-Feldman[7,8,9], Jared Feldman[1], Boris Julg[1], Eric J. Nilles[12], Elon R. Musk[3], Anil S. Menon[3], Eric S. Fischer[4,5,6], Jason S. McLellan[11], Aaron Schmidt[1], Marcia B. Goldberg[7,9,13,14#], Michael R. Filbin[6,7#], Nir Hacohen[7,8,9#], Douglas A Lauffenburger[2#], Galit Alter[1#]

[1]Ragon Institute of MGH, MIT, and Harvard, Cambridge, MA, USA. [2]Department of Biological Engineering, Massachusetts Institute of Technology, Cambridge, MA, USA. [3]Space Exploration Technologies Corp, Hawthorne, CA, USA [4]Department of Cancer Biology, Dana-Farber Cancer Institute, Boston, MA, USA [5]Department of Biological Chemistry and Molecular Pharmacology, Harvard Medical School, Boston, MA, USA [6]Department of Emergency Medicine, Massachusetts General Hospital, Boston, MA, USA [7]Broad Institute of Massachusetts Institute of Technology (MIT) and Harvard, Cambridge, MA, USA. [8]Massachusetts General Hospital Cancer Center, Department of Medicine, Massachusetts General Hospital, Boston, MA, USA [9]Department of Medicine, Harvard Medical School, Boston, MA, USA [10]Center for Immunology and Inflammatory Diseases, Department of Medicine, Massachusetts General Hospital, Boston, MA, USA [11]Department of Molecular Biosciences, The University of Texas at Austin, Austin, TX, USA [12]Brigham and Women's Hospital, Boston, MA, USA [13]Department of Microbiology, Harvard Medical School, Boston, MA, USA [14]Department of Immunology and Infectious Diseases, Harvard TH Chan School of Public Health, Boston, MA, USA [15]Division of Infectious Diseases, Department of Medicine, Massachusetts General Hospital, Boston, MA, USA [16]Division of Gastroenterology, Department of Medicine, Massachusetts General Hospital, Boston, MA, USA

*contributed equally

#Corresponding author. Email: marcia.goldberg@mgh.harvard.edu (M.B.G.); MFILBIN@mgh.harvard.edu (M.R.F.); NHACOHEN@mgh.harvard.edu (N.H.); lauffen@mit.edu (D.L.); galter@partners.org (G.A.)

The introduction of vaccines has inspired new hope in the battle against SARS-CoV-2. However, the emergence of viral variants, in the absence of potent antivirals, has left the world struggling with the uncertain nature of this disease. Antibodies currently represent the strongest correlate of immunity against SARS-CoV-2, thus we profiled the earliest humoral signatures in a large cohort of acutely ill (survivors and non-survivors) and mild or asymptomatic individuals with COVID-19. While a SARS-CoV-2-specific immune response evolved rapidly in survivors of COVID-19, non-survivors exhibited blunted and delayed humoral immune evolution, particularly with respect to S2-specific antibodies. Given the conservation of S2 across β-coronaviruses, we found the early development of SARS-CoV-2-specific immunity occurred in tandem with pre-existing common β-coronavirus OC43 humoral immunity in survivors, which was also selectively expanded in individuals that develop a paucisymptomatic infection. These data point to the importance of cross-coronavirus immunity as a correlate of protection against COVID-19.

INTRODUCTION

The relentless spread and unpredictable nature of disease caused by SARS-CoV-2 continues to paralyze the globe. However, the introduction of potent vaccines has inspired new hope that the end of the pandemic is in sight (*1*–*3*). Yet, the slow vaccine rollout, emergence of new viral variants (*4*–*6*), confusing results of convalescent plasma trials, incomplete efficacy from monoclonal therapeutics, coupled with the lack of potent antiviral therapeutics, has left the globe with a burden of managing the uncertain nature of this disease. Thus, there is an urgent and continued need to characterize the humoral antibody response to acute disease and its correlate with outcomes, to better define biomarkers to support clinical care and target the design of monoclonal antibody therapeutics strategies.

SARS-CoV-2 infected patients experience a wide range of clinical manifestations ranging from asymptomatic infection to severe disease that may exacerbate and result in acute respiratory distress syndrome (ARDS) and ultimately death (*7*). However, while age (*8*) and comorbidities have been linked to more severe disease (*9*–*12*), the outcome of SARS-CoV-2 infection is unpredictable. Emerging immune correlate analyses have suggested that early robust neutralizing antibody responses (*13*–*15*), innate immune responses (*16*), Fc-receptor

activity (*17*, *18*), as well as altered B cell and T cell frequencies (*19*, *20*) and viral loads (*21–23*) are all linked to differential outcome.

Among these emerging correlates, antibodies are implicated in both natural resolution of infection (*16*, *21*, *24*) and protection following vaccination (*25*, *26*) with antibody-mediated effector functions observed prior to the evolution of neutralizing antibody activity (*3*, *27*, *28*). Importantly, beyond neutralization, antibodies control or clear infection by leveraging the immune system via their constant domain (Fc) functions that evolve promptly following natural infection (*16*). Specifically, antibodies targeting the S2 domain, the most conserved region of the SARS-CoV-2 Spike, evolve earliest and predict survival of natural SARS-CoV-2 infection (*16*). Moreover, S2-specific neutralizing antibodies have been observed in plasma samples collected prior to the pandemic (*29*, *30*). Yet whether these responses emerge from pre-existing common coronavirus immune responses remains unclear.

Thus, using samples from a large acute SARS-CoV-2 infection study (*31*) we profiled the evolving humoral immune response to SARS-CoV-2 over the first twelve days following symptom onset and in a community based mild infection cohort. Class switched SARS-CoV-2 S2-specific humoral immune responses evolved rapidly and selectively in acute survivors of COVID-19. Moreover, IgM and IgG responses to the common β-coronavirus OC43 were significantly expanded in individuals that survived infection and experienced milder disease. Furthermore, the earliest OC43-specific responses were linked to the acutely evolving SARS-CoV-2 responses across the disease spectrum, pointing to the importance of leveraging pre-existing common-coronavirus immunity to control and ultimately clear the infection across the disease spectrum. Thus, rather than original antigenic sin, pre-existing cross-CoV immunity may accelerate the evolution of cross-reactive humoral immune responses to highly conserved regions of the virus, which may point to critical targets of CoV immunity.

RESULTS

Dampened SARS-CoV-2 humoral immune evolution is a signature of COVID-19 mortality

Previous studies have noted distinct humoral evolutionary trajectories across individuals with different clinical outcomes following SARS-CoV-2 infection, marked by different magnitudes of humoral immune responses (*16*, *32*), differential targeting of antigens (*21*), increased functional breadth (*32*), incomplete IgG class switching (*33*), or neutralization activity (*14*). However, many of these studies probed the humoral immune response several days to weeks following symptom onset. Thus, to gain insights into the earliest host-pathogen interactions that may underline differences in disease trajectory, we profiled a cohort of acutely ill COVID-19 patients. A total of 217 patients with confirmed SARS-CoV-2 infection by nasopharyngeal PCR were collected at the time of admission through the Emergency Department (ED) at approximately 0 - 12 days following symptom onset and stratified by disease severity and 28-day outcome into three groups: (group 1) moderate - requiring hospitalization and supplemental oxygen support (n = 118, corresponding to a max score of 4 on the WHO Ordinal Outcomes scale in 28 days); (group 2) severe – intubated survivors up to 28 days (n = 62, corresponding to max WHO scale of 6-7); and (group 3) deceased - non-survivor (n = 37, corresponding to WHO scale 8) (*31*, *34*) (**Fig. 1A, Table S1**).

System Serological profiling (**Fig. 1B**) during the acute window of infection pointed to a significant deficit in IgG1 RBD, full S protein, S2, and N-specific antibody, and S2-specific IgM levels in the non-survivor group between 3-9 days after symptom onset (**Fig. 1C**). Similar trends, were noted for SARS-CoV-2-specific IgG3 and IgA1 titers, as well as FcγR binding capacity (**Fig. S1**). Specifically, SARS-CoV-2-specific antibodies able to bind to low affinity FcRs (FcγR2A, FcγR2B, FcγR3A and FcγR3B) were generated at lower levels in non-survivors compared to COVID-19 survivors (**Fig. S1**). Moreover, while FcγR binding was associated with neutralization in survivors of COVID-19, this relationship was lost in individuals that ultimately passed away (**Fig. S2**). Overall, humoral immune resposnes were poorly coordinated in the non-survivors compared to individuals that experienced moderate or severe disease.

Given that antibodies are generated as polyclonal swarms, we next profiled the coordination of the evolution of the humoral immune response across the groups. Strikingly higher coordination was observed in severe disease survivors within 0-3 days of symptom onset compared to the limited coordination observed in the moderate and non-survivor groups (**Fig. 1D**), with higher overall levels of coordination in the severe survivors (**Fig. 1E**). Conversely, coordination in the humoral immune response increased at day 3-6 and immediately decreased in individuals that did not survive SARS-CoV-2 infection, but persisted in individuals with moderate disease. This analysis suggests that severe survivors of COVID-19 generate a more robust, highly coordinated acute humoral immune responses very early in disease compared to patients that succumbed to COVID-19.

S2-specific responses are selectively enriched in survivors of COVID-19

Considering the multitude of differences across the groups, we next aimed to define whether specific longitudinal humoral features could resolve survivors from non-survivors. Thus, we generated pairs of nested mixed linear models across each set of subjects, accounting for comorbidities, age, and sex, all attributes previously linked to more severe COVID-19 (*35*) (**Fig. 2A-C**). First, we compared the SARS-CoV-2 response across survivors with severe disease to

individuals that did not survive severe disease. Following correction for multiple comparisons, six features, including S2 FcγR2, S2 FcγR2B, S2 FcγR3A, S2 FcγR3B, S1 IgG2, and RBD FcγR3B, were selectively enriched among patients that survived COVID-19. No features were selectively enriched in non-survivors. Interestingly, the four features directed at the highly conserved S2-domain of the SARS-CoV-2 Spike included Fc-receptor binding activity, rather than antibody IgG titers (**Fig. 2A**), pointing to an early enrichment of functional antibodies largely directed at the S2-domain as a marker of survival.

In contrast, a more balanced distribution of antibody features was observed when comparing moderate and severe survivors, with solely S2-specific IgG4 levels enriched among severe survivors (**Fig. 2B**), again illustrating the critical value of S2-specific immunity as a marker of disease outcome. Finally, the comparison of survivors with moderate infection compared to non-survivors highlighted the presence of elevated immune responses in individuals with moderate disease, marked by five statistically significant S2-specific Fc-receptor binding antibody features that were selectively enriched among individuals with moderate disease compared to non-survivors (**Fig. 2C**). Collectively, these data highlight the enrichment of S2-specific Fc-profiles as early biomarkers of disease outcome, suggesting that these earliest responses may either represent biomarkers of mechanistic responses in the early control and clearance of infection required for survival of COVID-19.

Common coronavirus responses are enriched in survivors early in infection course

The near simultaneous evolution of S2-specific IgG and IgM at early time points in survivors (**Fig. S3),** displayed as a ratio of IgM/IgG levels (*13*, *22*, *36*–*38*), suggests either a remarkably rapid maturation of the humoral immune response, or the potential expansion of pre-existing cross-coronavirus immunity to the conserved S2-domain. Interestingly, the elevated IgM/IgG ratio observed in non-survivors points to a delay in class switching from IgM to IgG in this population (**Fig. S3**). Annual circulation of common coronaviruses (cCoV) gives rise to broad cross-coronavirus immunity. However, how this cCoV-specific immunity influences the disease trajectory of COVID-19 remains unclear (*39*–*46*). Among the cCoV, the β-coronavirus OC43 circulates annually in the USA (*47*). Thus, to begin to define the relationship between cCoV and SARS-CoV-2 outcomes, we profiled the OC43 specific humoral response across the groups. To avoid the detection of cross-reaction antibodies across OC43 and SARS-CoV-2, immune profiling focused on OC43 receptor binding domain (RBD) antibodies, due to the limited conservation of RBD domains across the β-coronaviruses. Unexpectedly, higher OC43 RBD-specific IgM and IgG1 levels were observed in survivors with severe and moderate disease, particularly within 3-6 days of symptom onset compared to non-survivors (**Fig. 3A**). Conversely, no differences were noted in IgA, IgG3, or Fc-receptor binding across the groups. Notably, only a slight increase was observed in all OC43 responses across the groups over the study period, indicating stability in cCoV immunity that was not boosted by infection. These data point to the early enrichment of OC43-immunity, but not an evolution of these responses, in COVID-19 survivors in the first week of observation.

To further define whether the presence of these differential OC43-specific immune responses could resolve the COVID-19 severity groups, we next integrated OC43 RBD-specific humoral immune profiles into the paired nested mixed linear models. While S2-specific humoral immune responses remained top predictors in severe survivors compared to non-survivors, OC43 RBD-specific IgM antibody levels were significantly and selectively also expanded in the severe survivors (**Fig. 3B**), pointing to the importance of early expanded OC43-IgM levels in resolving disease trajectory alongside the previously observed S2-specific Fc-binding signatures. Likewise, OC43 RBD-specific IgG1 was enriched in survivors with severe disease compared to moderate disease (**Fig. 3C**), pointing to the potential importance of more mature expanded OC43-response in the resolution of severe rather than moderate disease. Finally, OC43 RBD-IgM levels were the most discriminatory feature between individuals with moderate disease and individuals who died within 28 days (**Fig. 3D**), pointing again to the importance of leveraging pre-existing cCoV IgM immunity as a marker of early protective immunity against this respiratory pathogen (*48*, *49*). Thus these data suggest that the level of pre-existing cCoV immunity contributes early in disease to differentiating disease trajectory, with cCoV-IgM levels representing a determinant of survival.

To ultimately determine whether the magnitude of pre-existing cCoV immunity could be linked to the evolution of early SARS-CoV-2 immunity across groups, we next examined at the relationship between the earliest OC43- and SARS-CoV-2-responses across all groups over time. Negative correlations would indicate that pre-existing cCoV immune responses dampened or blocked the evolution of SARS-CoV-2 immunity and positive correlations would suggest that the pre-existing cCoV-immune response may give rise to evolving SARS-CoV-2 immunity. Striking differences were observed in the overall correlational structure of the earliest OC43-and SARS-CoV-2 responses (**Fig. 3E** and **F**). Importantly, no negative correlations were observed. Instead, enhanced positive correlations were observed across OC43/SARS-CoV-2 in survivors compared to non-survivors, marked by more correlations in survivors with moderate disease, followed by severe disease compared to non-survivors, pointing to the importance of rapidly evolving cross-reactive immunity as a

marker of enhanced disease control. Interestingly, relationships were observed across S, S1, S2, Nucleocapsid, and RBD, suggesting that pre-existing OC43 responses likely mark a more generally cross-reactive immune response, enabling broad SARS-CoV-2 specific humoral immune evolution across antigens. Conversely, these relationships decayed across all groups just three days later, possibly suggesting that this relationship was only observable early in infection, prior to the evolution and divergence of the rapidly evolving SARS-CoV-2 specific affinity matured immune response.

Expanded S2-specific Fc-receptor binding antibodies are selectively enriched in asymptomatic infection

To test whether cCoV-cross reactive immunity was solely a marker of enhanced disease control in hospitalized patients, or may also represent a marker of generally milder disease, we extended the analysis of cCoV immunity to a community based cohort study of asymptomatic/paucisymptomatic SARS-CoV-2 infection. Individuals were sampled both prior to and after SARS-CoV-2 infection (*50*). While all individuals harbored robust class switched IgA and IgG OC43 responses prior to infection, no differences were observed in OC43-specific antibody profile across individuals asymptomatic (level 0), individuals that evolved a single symptom (level 1), or few mild symptoms (level 2) **(Fig. S4)** prior to or after infection (**Fig. 4A**). Conversely, after infection, OC43-specific IgG1 were selectively increased in indiviuals that remained asymptomatic or a single symptom. These data argue that class switched OC43-specific immunity may also be expanded and linked to more robust containment of asymptomatic/paucisymptomatic SARS-CoV-2 disease. Similarly, robust SARS-CoV-2-specific humoral immune responses (IgM, IgA, IgG1, and IgG3) were noted in both asymptomatic and paucisymptomatic (symptoms level 1 and 2) individuals. However, an unexpected difference was noted in the Fc-receptor binding ability of SARS-CoV-2 specific antibodies, and specifically in S2-specific humoral immune responses across symptom levels. Specifically, while asymptomatic and individuals that only experienced a single symptom did not elicit S1 or RBD-specific antibodies able to bind to Fc-receptors, these unique individuals solely generated Fc-receptor binding S2-specific antibodies (**Fig. 4B**). At a more granular level, while S2-specific IgG1 humoral immune responses were expanded across individuals, at all symptoms levels, S2-specific antibodies with the capacity to bind to the phagocytic FcγR2A and cytotoxic FcγR3A receptors were elicited in asymptomatic individuals. Conversely, all SARS-CoV-2 specific antibodies had the capacity to interact with Fc-receptors in individuals with more symptoms following mild SARS-CoV-2 infection. Together, these data suggest that the expansion of S2-specific responses may emerge rapidly following infection, derived from pre-existing cCoV immunity, and contribute to disease seveirty via Fc-mediated antiviral control and clearance.

DISCUSSION

Since its idenfication in late 2019, SARS-CoV-2 has caused hundreds of millions of infections, more than four million deaths, overwhelmed health systems, and impacted global economies (*51*, *52*). Public health measures, including masks, distancing, quarantines, have helped to slow the spread of the virus (*53*, *54*), but the rapid evolution of variants of concern, coupled to the relaxation of public health measures, has led to global increases in spread. While SARS-CoV-2 specific therapeutics have shown more moderate promise (*55*), vaccines are likely to be key to ending the pandemic. However, with the rise of SARS-CoV-2 variants that evade vaccine mediated neutralization, the development of next-generation vaccines or boosting strategies has been difficult in the absence of precise correlates of immunity against COVID-19.

However, beyond neutralization, Fc-effector functions have been linked to the resolution of natural infection (*56*–*60*) and vaccine-induced protection (*4*, *61*, *62*). Moreover, Fc-effector functions have also been mechanistically implicated in the efficacy of particular monoclonal therapeutics against SARS-CoV-2 (*63*–*66*). Moreover, early spike-specific Fc-effector functions, mainly targeting the S2, were previously observed among survivors (*16*, *21*) and pre-pandemic-S2-specific neutralizing antibodies have been observed primarily in children (*29*) that are typically spared from COVID-19 (*67*). However, the precise origin and specificity of these early and/or pre-existing S2-reactive antibodies were unclear. Here, using acute hospitalized COVID-19 patients, an association was observed between Fc-receptor binding S2-specific humoral immune responses and survival COVID-19. Specifically, S2 class-switched IgG antibodies, with the capaibiltiy of binding to multiple Fc-receptors, were observed just days after symptom onset, pointing to the rapid emergence of SARS-CoV-2 functional humora immunity, likely from some pre-existing humoral reservoir, rather than solely attributable to de novo evolution of these responses. The observation concurs with previous data, substantiating the earliest evolution of S2-specific immunity, with other specificities evolving more slowly, in an orthogonal cohort (*16*). Given the conserved nature of S2 across β-coronaviruses, these data suggested that individuals who survive COVID-19 may have an earlier advantage as they may be able to rapidly redeploy S2-specific antibodies across coronaviruses, to combat disease.

Unlike the potent neutralizing activity of antibodies against RBD, S2-specific antibodies are more weakly neutralizing (*68*–*70*), and thus likely provide protection through additional humoral mechanisms (*16*). Like the influenza stem-specific antibodies that contribute to protection via Fc-dependent antibody dependent cellular cytotoxicity (*71*), recent work in animals highlights the importance of Fc-dependent mechanisms for S2-specific monoclonal antibodies (*69*). In

our cohort of acute COVID-19 patients, S2-specific antibodies with Fc-receptor binding capabilities, rather than S2-titers alone, were among the strongest correlates of protective immunity against death. Moreover, S2-specific antibody functions were selectively augmented in asymptomatic SARS-CoV-2 infection, suggesting that the ability of these antibodies to recruit specific innate immune effector functions may be key to their protective activity. Thus, even in the absence of potent neutralization, S2-specific antibody effector functions may be key to the early recognition, control, and clearance of viruses resulting in attenuated disease. Thus, unlike RBD-specific antibodies that may be critical for driving sterilizing immunity, S2-specific antibodies may be key to attenuating disease (*69*). Moreover, given the highly conserved nature of S2, it is plausible that highly functional S2-specific monoclonal antibodies or vaccine-induced immune responses may provide broader and more potent protection against emerging SARS-CoV-2 variants and perhaps even additional β-coronaviruses. Thus, despite the increasing numbers of infections observed globally in the wake of the emergence of the highly contagious Delta (B.1.617.2) variant, rates of hospitalization have not increased proportionally (*72*). Whether this protection is related to the ability of vaccines to drive robust S2-specific immune responses remains unclear, but could account for high levels of breakthroughs in the absence of disease.

S2-specific responses exhibit delayed kinetics in non-survivors compared to severe and moderate survivors of COVID-19. Thus, while current RBD-specific monoclonal therapeutics have struggled to compete with pre-existing S1/RBD specific antibodies that were already present in the circulation of severely ill individuals (*73*), it is plausible that the administration of highly functional antibodies to a less immunodominant target in vulnerable populations could represent a critical opportunity to improve disease outcomes. Thus, the delivery of S2-specific monoclonal antibodies may offer an opportunity to supplement a missing population of antibodies to support the response to SARS-CoV-2 across a broader clinical window of therapeutic opportunity to control and clear the infection. Hency, identification of the most potent S2-antibodies, linked to key antibody effector function, may shift the host-pathogen interaction and drive enhanced protective immunity.

The presence of higher acute OC43 specific humoral immune response among patients with better clinical outcomes raised the possibility that cross-reactive SARS-CoV-2 specific immunity may emerge from pre-existing OC43-specific humoral memory. Unexpectedly, OC43 specific IgG and IgM antibodies were among the top discriminatory features in survivors. Specifically, OC43-specific IgG was an acute marker of survival of severe disease, whereas OC43-specific IgM response was a marker of moderate disease. While traditionally, IgM is considered a marker of a newly emerging immune response, mounting data suggest that IgM responses can persist throughout infection, continue to affinity mature, and remerge to fight infection from memory (*48*, *49*, *74*). For example, some of the most potent universal Influenza specific antibodies have been cloned from affinity matured IgM+ memory B cells (*74*, *75*), pointing to the importance of IgM-memory as a critical source of potentially protective antibodies in case of respiratory infections. Importantly, given the remarkable avidity and functional potency of IgM, able to drive robust complement and opsonophagocytic activity (*76*–*78*), it is plausible that IgM responses may be sufficient in the context of acute infection to drive rapid control and clearance of the pathogen. Moreover, SARS-CoV-2 specific IgM monoclonal therapeutics are more potent than IgG therapeutics in the treatment of SARS-CoV-2 infection in animal models (*79*–*81*), potentially pointing to the possibility that individuals able to selectively expand their OC43-specific IgM or IgG will respond better to COVID-19.

Importantly we did not see any interference- or evidence of antigenic imprint shaped by the presence of pre-existing cross-coronavirus immunity. Thus unlike, original antigenic sin in the context of influenza where pre-existing immunity to a particular strain of influenza prevents boosting to orthogonal contemporaneous strains (*82*), we did not observe any evidence of anti-correlated SARS-CoV2 immunity depending on the magnitude or quality of the pre-existing OC43-specific cCoV immune response. We observed global coordination of SARS-CoV–specific humoral immune responses with OC43 in survivors, albeit these relationships did not exist or were subdued in non-survivors. Because the RBD and S1 share limited homology across the viruses, it is less likely that pre-existing responses to OC43 could directly lead to the evolution of S1- or RBD-specific responses. However, the presence of class-switched OC43-specific humoral immunity is likely accompanied by a robust cCoV-T cell immune response across the viral proteome, that is likely to also play a key part in the accelerated evolution of the humoral immune response across additional conserved antigenic targets including S2 and the nucleocapsid (N). Thus, whether S2-antibodies alone or pre-existing cross-coronavirus specific T cells contribute to enhanced control of acute infection remains unclear, but points to a potential intersection between the humoral and cellular immune response, to conserved sites, that may be key to protective immunity against SARS-CoV-2.

Collectively, despite our inability to capture individuals prior to symptom onset, our study provided an opportunity to map humoral immune resposnes from nearly the time of symptom onset that tracked with significantly different clinical outcomes. Deep humoral profiling pointed to the presence of an acute S2-specific Fc-receptor binding signature as

a marker of survival of disease and reduced symptomatology, likely evolving from early pre-existing robust cCoV humoral immunity. While these S2-specific humoral immune responses are likely to permit breakthrough infections, the development of future vaccines or boosting regimens able to promote immunity to this highly conserved domain of SARS-CoV-2 may provide broad protection against emerging variants of concern and even other coronaviruses.

METHODS

Study design

The objective of the study was to define humoral profiles that could predict differences in clinical trajectory following SARS-CoV-2 infection. The study began in March 2020, focused on comparing acutely ill COVID-19 patients presenting to the emergency room at the Massachusetts General Hospital (MGH) or from a community based surveillance study run through Space Exploration Technologies Corporation. Acute subjects were categorized by the clinical team. This study was blinded until the final analysis. All experiments were performed in technical replicates. The study protocol was approved by MGH and the Western Institutional Review Board. All participants provided written informed consent.

Patient cohort and clinical data collection

Acutely ill COVID-19 patients: Patients 18 years or older (n = 384) with acute respiratory distress and clinical concern for COVID-19 were enrolled in the Emergency Department (ED) in Boston during the peak of the COVID-19 surge (from 3/24/2020 to 4/30/2020), 306 of whom tested positive for SARS-CoV-2 nasopharyngeal PCR as described by Filbin *et al.* (*31*). This analysis included SARS-CoV-2-positive patients whose symptoms onset was between 0-12 days prior to presentation and whose illness severity and 28-day outcome were classified into three groups; (group 1) moderate – hospitalized and requiring oxygen support but not mechanical ventilation (n = 118, corresponding to WHO Ordinal Outcomes scale 4); (group 2) severe – intubated but survived to 28 days (n = 62, corresponding to WHO scale 6-7); and (group 3) deceased within 28 days - non-survivor (n = 37, corresponding to WHO scale 8) (*34*). Of the 42 COVID-19 patients who died, 24 (57%) received mechanical ventilation, and 18 (43%) did not. Patients were excluded from analysis if they were discharged directly from the ED and were not hospitalized within the next 28 days, or if they were admitted but did not require supplemental oxygen.

Day 0 blood samples were obtained with the initial clinical blood draw in the ED, and day three and day seven samples were obtained during patients' hospitalization. The clinical course was followed for 28 days post-enrollment to establish outcomes. Samples and clinical information were collected according to an institutional IRB-approved protocol (*31*). Symptom duration upon presentation was obtained via chart review. Demographic, medical history, and clinical data were collected and summarized for each outcome group, using medians with interquartile ranges and proportions with 95% confidence intervals, where appropriate.

Community-acquired mild and asymptomatic COVID-19 individuals: Industry employees (Space Exploration Technologies Corp.) included volunteers tested for COVID-19 starting in mid-April 2020. All employees were invited to participate by email, and there were no exclusion criteria. Participants completed a study survey including the collection of COVID-19 related symptoms (*50*). Upon obtaining informed consent, blood samples were collected approximately every 39.7 days (standard deviation 13.8 days). Symptoms were classified by severity, with 2 points being assigned to loss of smell/taste, fever, feverish/chills, or cough, and 1 point being assigned to other symptoms such as increased fatigue, headache, congestion, nausea/vomiting, diarrhea, sore throat, and body/muscle aches. Symptom scores were summed and each subject was categorized into one of three levels based on degree of symptoms: level 0 (n = 18), no symptoms; level 1 (n = 27), mild, symptom score 1-5; level 2 (n = 13), moderate, symptom score 6-14.

Luminex

SARS-CoV-2 and eCoV-specific antibody subclass/isotype and Fcγ-receptor (FcγR) binding levels were assessed using a 384-well based customized multiplexed Luminex assay, as previously described (*83*). SARS-CoV-2 receptor binding domain (RBD) (kindly provided by Aaron Schmidt, Ragon Institute), SARS-CoV-2 nucleocapsid (N) protein (Aalto BioReagents), and SARS-CoV-2 spike protein (S) (kindly provided by Eric Fischer, Dana Farber), SARS-CoV-2 subunit 1 and 2 of the spike protein (S1 and S2) (Sino Biological), as well as human eCoV antigens: hCoV-OC43 RBD (kindly provided by Aaron Schmidt, Ragon Institute), hCoV-OC43 spike protein (S) (Sino Biological), hCoV-HKU1 spike protein (S) (Immune Tech), SARS-CoV-1, MERS spike proteins (S) (kindly provided by Jason McLellan, University of Texas) were used to profile specific humoral immune responses. A mix of HA A/Michigan/45/2015 (H1N1), HA A/Singapore/INFIMH-16-0019/2016 (H3N2), B/Phuket/3073/2013 (Immunetech) was used as a control. Antigens were coupled to magnetic Luminex beads (Luminex Corp) by carbodiimide-NHS ester-coupling (Thermo Fisher). Antigen-coupled microspheres were washed and incubated with plasma samples at an appropriate sample dilution (1:500 for IgG1 and all Fcγ-receptors, and 1:100 for all other readouts) for 2 hours at 37°C in 384-well plates (Greiner Bio-One). Unbound antibodies were washed away, and antigen-bound antibodies were detected by using a PE-coupled detection antibody for each subclass and isotype (IgG1, IgG2, IgG3, IgG4, IgA1, and IgM; Southern Biotech), and Fcγ-receptors were fluorescently labeled with PE before addition to immune complexes (FcγR2A, FcγR2B, FcγR3A, FcγR3B; Duke Protein

Production facility). After one h incubation, plates were washed, and flow cytometry was performed with an IQue (Intellicyt), and analysis was performed on IntelliCyt ForeCyt (v8.1). PE median fluorescent intensity (MFI) is reported as a readout for antigen-specific antibody titers.

Quantification and Statistical Analysis:

All analyses were performed using R version 4.0.3. All the figures were created with many R-supported packages, mainly including ggplot, ggrepel, ggpubr.

Data Pre-processing

The raw MFI (median florescent intensity) from flow cytometry was scaled by the log10 function. To control for noise, PBS values were subtracted from the scaled titer measurements.

Univariate Plots

The box-plots summarize the median value with first and third quantiles for each clinical group (moderate, severe, and deceased) across day ranges from symptom onset within an interval of three days (day ranges 0-3, 3- 6, 6- 9, 9- 12). Paired *p*-value were defined by the Mann-Whitney U test for each sub-feature on the individual temporal course across different clinical groups and adjusted by the Benjamini-Hochbery procedure of multiple testing correction. The visualization was performed by the function 'ggplot' of R package 'ggplot2' (3.3.3), and the p-value was estimated by the function 'wilcox_test' and 'adjust_pvalue' in the R package 'rstatix'(0.6.0) and labeled by the function 'stat_pvalue_manual' in the R packge 'ggpubr' (0.4.0).

Correlation Analysis

Spearman correlations were used to evaluate the relationship between different measurements and were performed using the function 'rcorr' of R package 'Hmisc' (4.4.2). A time-specific correlation analysis between different antibody measurements was used to explore temporal coordination between features. Specifically, Spearman coefficients were defined between titer values across timepoints within each clinical group. The significance of correlation was adjusted by the Benjamini-Hochbery procedure of multiple testing correction.

To avoid the impact of differering sample sizes, we evaluated the effect of different sample sizes across disease severities and time intervals, two strategies were applied: 1) First, for the purpose of visualization, correlation coefficients were considered only if larger than 0.6, given that coefficients may reach significance more easily using larger sample sizes; 2) A downsampling strategy was also explored to equalize the sample-size across different groups for statistical evaluation. For the down-sampling, we randomly downsampled ten samples and calculated the Spearman correlation for 500 random-sampling runs. Then, the number of significant correlations larger than 0.6 were calculated and tested by the Mann-Whitney U test and multiple testing correction was implemented to avoid statistical anomalies.

Defining signatures of disease outcome while also controlling for potential cofounders

We accessed the significance of the association between measured antibody levels and clinical outcomes by controlling for collected potential cofounders, using two nested mixed linear models (null and full model) without/with clinical outcomes. We fit two linear mixed models and estimated the improvement in model fit by likelihood ratio testing to assess how many measurements have a significantly better fit with the full model at a threshold of < 0.05.

Null model:

$$\begin{aligned} &antibody\ measurement \sim TimeFactor + well.plate + \\ &physical.information + historical.diseases + \\ &TimeFactor*Symptom + TimeFactor* \\ &immunemeasurement + (1 | Pat) \end{aligned}$$

Full model:

$$\begin{aligned} &antibody\ measurement \sim TimeFactor + well.plate + \\ &physical.information + \mathbf{Clinical.outcome} + \\ &historical.disease + TimeFactor*Symptom + \\ &TimeFactor*immune.measurement + (1 | Pat) \end{aligned}$$

Likelihood Ratio Test:

$$LRT = -2*\ln\left(\frac{MLE\ in\ Full\ model}{MLT\ in\ Null\ model}\right) \sim \lambda^2$$

Here, the *historical.diseases* include comorbidities such as heart, lung, kidney, and diabetes, while *physical.information* included age and BMI. *Well.plate* represented the batch indicator on the Luminex platform. *Time-related* symptoms included respiratory symptom, fever, gastrointestinal symptoms, and other immune measurements, such as absolute neutrophil count, absolute monocyte count, creatinine, the level of C-reactive protein (CRP), d-dimer, lactate dehydrogenase (LDH) along the time, and troponin level at 72 hours. The R package 'lme4' was used to fit the linear mixed model to each measurement and test for measurement across the contrast of interest. The *p*-value from the Likelihood Ratio test and t value of Clinical outcome in Full model was visualized as volcano plot using the 'ggplot' function in R package 'ggplot2'.

Statistics

Statistical analyses were performed using R ggpubr and ggcorrplot packages. Statistical significance between groups was determined using two-sided Mann-Whitney tests to compare ranks implemented in the *wilcox_test* function of R rstatix package. *P* values <0.05 were considered significant. Additionally, the p values were adjusted by Benjamini-Hochberg multiple testing correction.

SUPPLEMENTARY MATERIALS

www.science.org/doi/10.1126/sciimmunol.abj2901
Figs. S1 to S4
Tables S1 and S2

REFERENCES AND NOTES

1. L. A. Jackson, E. J. Anderson, N. G. Rouphael, P. C. Roberts, M. Makhene, R. N. Coler, M. P. McCullough, J. D. Chappell, M. R. Denison, L. J. Stevens, A. J. Pruijssers, A. McDermott, B. Flach, N. A. Doria-Rose, K. S. Corbett, K. M. Morabito, S. O'Dell, S. D. Schmidt, P. A. Swanson 2nd, M. Padilla, J. R. Mascola, K. M. Neuzil, H. Bennett, W. Sun, E. Peters, M. Makowski, J. Albert, K. Cross, W. Buchanan, R. Pikaart-Tautges, J. E. Ledgerwood, B. S. Graham, J. H. Beigel; mRNA-1273 Study Group, An mRNA Vaccine against SARS-CoV-2 - Preliminary Report. *N. Engl. J. Med.* **383**, 1920–1931 (2020). doi:10.1056/NEJMoa2022483 Medline
2. M. D. Knoll, C. Wonodi, Oxford-AstraZeneca COVID-19 vaccine efficacy. *Lancet* **397**, 72–74 (2021). doi:10.1016/S0140-6736(20)32623-4 Medline
3. F. P. Polack, S. J. Thomas, N. Kitchin, J. Absalon, A. Gurtman, S. Lockhart, J. L. Perez, G. Pérez Marc, E. D. Moreira, C. Zerbini, R. Bailey, K. A. Swanson, S. Roychoudhury, K. Koury, P. Li, W. V. Kalina, D. Cooper, R. W. Frenck Jr., L. L. Hammitt, Ö. Türeci, H. Nell, A. Schaefer, S. Ünal, D. B. Tresnan, S. Mather, P. R. Dormitzer, U. Şahin, K. U. Jansen, W. C. Gruber; C4591001 Clinical Trial Group, Safety and Efficacy of the BNT162b2 mRNA Covid-19 Vaccine. *N. Engl. J. Med.* **383**, 2603–2615 (2020). doi:10.1056/NEJMoa2034577 Medline
4. J. Klingler *et al.*, SARS-CoV-2 mRNA vaccines induce a greater array of spike-specific antibody isotypes with more potent complement binding capacity than natural infection. medRxiv. 2021:2021.05.11.21256972.
5. R. E. Chen, X. Zhang, J. B. Case, E. S. Winkler, Y. Liu, L. A. VanBlargan, J. Liu, J. M. Errico, X. Xie, N. Suryadevara, P. Gilchuk, S. J. Zost, S. Tahan, L. Droit, J. S. Turner, W. Kim, A. J. Schmitz, M. Thapa, D. Wang, A. C. M. Boon, R. M. Presti, J. A. O'Halloran, A. H. J. Kim, P. Deepak, D. Pinto, D. H. Fremont, J. E. Crowe Jr., D. Corti, H. W. Virgin, A. H. Ellebedy, P.-Y. Shi, M. S. Diamond, Resistance of SARS-CoV-2 variants to neutralization by monoclonal and serum-derived polyclonal antibodies. *Nat. Med.* **27**, 717–726 (2021). doi:10.1038/s41591-021-01294-w Medline
6. S. A. Kemp *et al.*, Neutralising antibodies in Spike mediated SARS-CoV-2 adaptation. medRxiv. 2020.
7. A. Gupta, M. V. Madhavan, K. Sehgal, N. Nair, S. Mahajan, T. S. Sehrawat, B. Bikdeli, N. Ahluwalia, J. C. Ausiello, E. Y. Wan, D. E. Freedberg, A. J. Kirtane, S. A. Parikh, M. S. Maurer, A. S. Nordvig, D. Accili, J. M. Bathon, S. Mohan, K. A. Bauer, M. B. Leon, H. M. Krumholz, N. Uriel, M. R. Mehra, M. S. V. Elkind, G. W. Stone, A. Schwartz, D. D. Ho, J. P. Bilezikian, D. W. Landry, Extrapulmonary manifestations of COVID-19. *Nat. Med.* **26**, 1017–1032 (2020). doi:10.1038/s41591-020-0968-3 Medline
8. K. Liu, Y. Chen, R. Lin, K. Han, Clinical features of COVID-19 in elderly patients: A comparison with young and middle-aged patients. *J. Infect.* **80**, e14–e18 (2020). doi:10.1016/j.jinf.2020.03.005 Medline
9. M. S. Suthar, M. G. Zimmerman, R. C. Kauffman, G. Mantus, S. L. Linderman, W. H. Hudson, A. Vanderheiden, L. Nyhoff, C. W. Davis, O. Adekunle, M. Affer, M. Sherman, S. Reynolds, H. P. Verkerke, D. N. Alter, J. Guarner, J. Bryksin, M. C. Horwath, C. M. Arthur, N. Saakadze, G. H. Smith, S. Edupuganti, E. M. Scherer, K. Hellmeister, A. Cheng, J. A. Morales, A. S. Neish, S. R. Stowell, F. Frank, E. Ortlund, E. J. Anderson, V. D. Menachery, N. Rouphael, A. K. Mehta, D. S. Stephens, R. Ahmed, J. D. Roback, J. Wrammert, Rapid Generation of Neutralizing Antibody Responses in COVID-19 Patients. *Cell Rep Med.* **1**, 100040 (2020). doi:10.1016/j.xcrm.2020.100040 Medline
10. M. Zuin, G. Rigatelli, G. Zuliani, A. Rigatelli, A. Mazza, L. Roncon, Arterial hypertension and risk of death in patients with COVID-19 infection: Systematic review and meta-analysis. *J. Infect.* **81**, e84–e86 (2020). doi:10.1016/j.jinf.2020.03.059 Medline
11. F. Zhou, T. Yu, R. Du, G. Fan, Y. Liu, Z. Liu, J. Xiang, Y. Wang, B. Song, X. Gu, L. Guan, Y. Wei, H. Li, X. Wu, J. Xu, S. Tu, Y. Zhang, H. Chen, B. Cao, Clinical course and risk factors for mortality of adult inpatients with COVID-19 in Wuhan, China: A retrospective cohort study. *Lancet* **395**, 1054–1062 (2020). doi:10.1016/S0140-6736(20)30566-3 Medline
12. W. J. Guan, W. H. Liang, Y. Zhao, H. R. Liang, Z. S. Chen, Y. M. Li, X. Q. Liu, R. C. Chen, C. L. Tang, T. Wang, C. Q. Ou, L. Li, P. Y. Chen, L. Sang, W. Wang, J. F. Li, C. C. Li, L. M. Ou, B. Cheng, S. Xiong, Z. Y. Ni, J. Xiang, Y. Hu, L. Liu, H. Shan, C. L. Lei, Y. X. Peng, L. Wei, Y. Liu, Y. H. Hu, P. Peng, J. M. Wang, J. Y. Liu, Z. Chen, G. Li, Z. J. Zheng, S. Q. Qiu, J. Luo, C. J. Ye, S. Y. Zhu, L. L. Cheng, F. Ye, S. Y. Li, J. P. Zheng, N. F. Zhang, N. S. Zhong, J. X. He; China Medical Treatment Expert Group for COVID-19, Comorbidity and its impact on 1590 patients with COVID-19 in China: A nationwide analysis. *Eur. Respir. J.* **55**, 2000547 (2020). doi:10.1183/13993003.00547-2020 Medline
13. Q.-X. Long, B.-Z. Liu, H.-J. Deng, G.-C. Wu, K. Deng, Y.-K. Chen, P. Liao, J.-F. Qiu, Y. Lin, X.-F. Cai, D.-Q. Wang, Y. Hu, J.-H. Ren, N. Tang, Y.-Y. Xu, L.-H. Yu, Z. Mo, F. Gong, X.-L. Zhang, W.-G. Tian, L. Hu, X.-X. Zhang, J.-L. Xiang, H.-X. Du, H.-W. Liu, C.-H. Lang, X.-H. Luo, S.-B. Wu, X.-P. Cui, Z. Zhou, M.-M. Zhu, J. Wang, C.-J. Xue, X.-F. Li, L. Wang, Z.-J. Li, K. Wang, C.-C. Niu, Q.-J. Yang, X.-J. Tang, Y. Zhang, X.-M. Liu, J.-J. Li, D.-C. Zhang, F. Zhang, P. Liu, J. Yuan, Q. Li, J.-L. Hu, J. Chen, A.-L. Huang, Antibody responses to SARS-CoV-2 in patients with COVID-19. *Nat. Med.* **26**, 845–848 (2020). doi:10.1038/s41591-020-0897-1 Medline
14. W. F. Garcia-Beltran, E. C. Lam, M. G. Astudillo, D. Yang, T. E. Miller, J. Feldman, B. M. Hauser, T. M. Caradonna, K. L. Clayton, A. D. Nitido, M. R. Murali, G. Alter, R. C. Charles, A. Dighe, J. A. Branda, J. K. Lennerz, D. Lingwood, A. G. Schmidt, A. J. Iafrate, A. B. Balazs, COVID-19-neutralizing antibodies predict disease severity and survival. *Cell* **184**, 476–488.e11 (2021). doi:10.1016/j.cell.2020.12.015 Medline
15. A. S. Iyer *et al.*, Dynamics and significance of the antibody response to SARS-CoV-2 infection. medRxiv. 2020.
16. T. Zohar, C. Loos, S. Fischinger, C. Atyeo, C. Wang, M. D. Slein, J. Burke, J. Yu, J. Feldman, B. M. Hauser, T. Caradonna, A. G. Schmidt, Y. Cai, H. Streeck, E. T. Ryan, D. H. Barouch, R. C. Charles, D. A. Lauffenburger, G. Alter, Compromised Humoral Functional Evolution Tracks with SARS-CoV-2 Mortality. *Cell* **183**, 1508–1519.e12 (2020). doi:10.1016/j.cell.2020.10.052 Medline
17. C. Loos, C. Atyeo, S. Fischinger, J. Burke, M. D. Slein, H. Streeck, D. Lauffenburger, E. T. Ryan, R. C. Charles, G. Alter, Evolution of Early SARS-CoV-2 and Cross-Coronavirus Immunity. *MSphere* **5**, e00622-20 (2020). doi:10.1128/mSphere.00622-20 Medline
18. K. K. Q. Yu *et al.*, T cell and antibody functional correlates of severe COVID-19. medRxiv. 2020.
19. D. Mathew, J. R. Giles, A. E. Baxter, D. A. Oldridge, A. R. Greenplate, J. E. Wu, C. Alanio, L. Kuri-Cervantes, M. B. Pampena, K. D'Andrea, S. Manne, Z. Chen, Y. J. Huang, J. P. Reilly, A. R. Weisman, C. A. G. Ittner, O. Kuthuru, J. Dougherty, K. Nzingha, N. Han, J. Kim, A. Pattekar, E. C. Goodwin, E. M. Anderson, M. E. Weirick, S. Gouma, C. P. Arevalo, M. J. Bolton, F. Chen, S. F. Lacey, H. Ramage, S. Cherry, S. E. Hensley, S. A. Apostolidis, A. C. Huang, L. A. Vella, M. R. Betts, N. J. Meyer, E. J. Wherry; UPenn COVID Processing Unit, Deep immune profiling of COVID-19 patients reveals distinct immunotypes with therapeutic implications. *Science* **369**, eabc8511 (2020). doi:10.1126/science.abc8511 Medline
20. G. Chen, D. Wu, W. Guo, Y. Cao, D. Huang, H. Wang, T. Wang, X. Zhang, H. Chen, H. Yu, X. Zhang, M. Zhang, S. Wu, J. Song, T. Chen, M. Han, S. Li, X. Luo, J. Zhao, Q. Ning, Clinical and immunological features of severe and moderate coronavirus disease 2019. *J. Clin. Invest.* **130**, 2620–2629 (2020). doi:10.1172/JCI137244 Medline
21. C. Atyeo, S. Fischinger, T. Zohar, M. D. Slein, J. Burke, C. Loos, D. J. McCulloch, K. L. Newman, C. Wolf, J. Yu, K. Shuey, J. Feldman, B. M. Hauser, T. Caradonna, A. G. Schmidt, T. J. Suscovich, C. Linde, Y. Cai, D. Barouch, E. T. Ryan, R. C. Charles, D. Lauffenburger, H. Chu, G. Alter, Distinct Early Serological Signatures Track with SARS-CoV-2 Survival. *Immunity* **53**, 524–532.e4 (2020). doi:10.1016/j.immuni.2020.07.020 Medline
22. H. Luo, T. Jia, J. Chen, S. Zeng, Z. Qiu, S. Wu, X. Li, Y. Lei, X. Wang, W. Wu, R. Zhang, X. Zou, T. Feng, R. Ding, Y. Zhang, Y.-Q. Chen, C. Sun, T. Wang, S. Fang, Y. Shu, The Characterization of Disease Severity Associated IgG Subclasses Response in COVID-19 Patients. *Front. Immunol.* **12**, 632814 (2021). doi:10.3389/fimmu.2021.632814 Medline
23. Y. Wang, L. Zhang, L. Sang, F. Ye, S. Ruan, B. Zhong, T. Song, A. N. Alshukairi, R. Chen, Z. Zhang, M. Gan, A. Zhu, Y. Huang, L. Luo, C. K. P. Mok, M. M. Al Gethamy, H. Tan, Z. Li, X. Huang, F. Li, J. Sun, Y. Zhang, L. Wen, Y. Li, Z. Chen, Z. Zhuang, J. Zhuo, C. Chen, L. Kuang, J. Wang, H. Lv, Y. Jiang, M. Li, Y. Lin, Y. Deng, L. Tang, J. Liang, J. Huang, S. Perlman, N. Zhong, J. Zhao, J. S. Malik Peiris, Y. Li, J. Zhao, Kinetics of viral load and antibody response in relation to COVID-19 severity. *J.*

Clin. Invest. **130**, 5235–5244 (2020). doi:10.1172/JCI138759 Medline
24. D. F. Robbiani, C. Gaebler, F. Muecksch, J. C. C. Lorenzi, Z. Wang, A. Cho, M. Agudelo, C. O. Barnes, A. Gazumyan, S. Finkin, T. Hägglöf, T. Y. Oliveira, C. Viant, A. Hurley, H.-H. Hoffmann, K. G. Millard, R. G. Kost, M. Cipolla, K. Gordon, F. Bianchini, S. T. Chen, V. Ramos, R. Patel, J. Dizon, I. Shimeliovich, P. Mendoza, H. Hartweger, L. Nogueira, M. Pack, J. Horowitz, F. Schmidt, Y. Weisblum, E. Michailidis, A. W. Ashbrook, E. Waltari, J. E. Pak, K. E. Huey-Tubman, N. Koranda, P. R. Hoffman, A. P. West Jr., C. M. Rice, T. Hatziioannou, P. J. Bjorkman, P. D. Bieniasz, M. Caskey, M. C. Nussenzweig, Convergent antibody responses to SARS-CoV-2 in convalescent individuals. *Nature* **584**, 437–442 (2020). doi:10.1038/s41586-020-2456-9 Medline
25. Z. Wang, F. Schmidt, Y. Weisblum, F. Muecksch, C. O. Barnes, S. Finkin, D. Schaefer-Babajew, M. Cipolla, C. Gaebler, J. A. Lieberman, T. Y. Oliveira, Z. Yang, M. E. Abernathy, K. E. Huey-Tubman, A. Hurley, M. Turroja, K. A. West, K. Gordon, K. G. Millard, V. Ramos, J. Da Silva, J. Xu, R. A. Colbert, R. Patel, J. Dizon, C. Unson-O'Brien, I. Shimeliovich, A. Gazumyan, M. Caskey, P. J. Bjorkman, R. Casellas, T. Hatziioannou, P. D. Bieniasz, M. C. Nussenzweig, mRNA vaccine-elicited antibodies to SARS-CoV-2 and circulating variants. *Nature* **592**, 616–622 (2021). doi:10.1038/s41586-021-03324-6 Medline
26. M. J. Gorman *et al*., Collaboration between the Fab and Fc contribute to maximal protection against SARS-CoV-2 in nonhuman primates following NVX-CoV2373 subunit vaccine with Matrix-M™ vaccination. bioRxiv. 2021.
27. D. M. Skowronski, G. De Serres, Safety and Efficacy of the BNT162b2 mRNA Covid-19 Vaccine. *N. Engl. J. Med.* **384**, 1576–1577 (2021). doi:10.1056/NEJMc2036242 Medline
28. F. Krammer, K. Srivastava, H. Alshammary, A. A. Amoako, M. H. Awawda, K. F. Beach, M. C. Bermúdez-González, D. A. Bielak, J. M. Carreño, R. L. Chernet, L. Q. Eaker, E. D. Ferreri, D. L. Floda, C. R. Gleason, J. Z. Hamburger, K. Jiang, G. Kleiner, D. Jurczyszak, J. C. Matthews, W. A. Mendez, I. Nabeel, L. C. F. Mulder, A. J. Raskin, K. T. Russo, A. T. Salimbangon, M. Saksena, A. S. Shin, G. Singh, L. A. Sominsky, D. Stadlbauer, A. Wajnberg, V. Simon, Antibody Responses in Seropositive Persons after a Single Dose of SARS-CoV-2 mRNA Vaccine. *N. Engl. J. Med.* **384**, 1372–1374 (2021). doi:10.1056/NEJMc2101667 Medline
29. K. W. Ng, N. Faulkner, G. H. Cornish, A. Rosa, R. Harvey, S. Hussain, R. Ulferts, C. Earl, A. G. Wrobel, D. J. Benton, C. Roustan, W. Bolland, R. Thompson, A. Agua-Doce, P. Hobson, J. Heaney, H. Rickman, S. Paraskevopoulou, C. F. Houlihan, K. Thomson, E. Sanchez, G. Y. Shin, M. J. Spyer, D. Joshi, N. O'Reilly, P. A. Walker, S. Kjaer, A. Riddell, C. Moore, B. R. Jebson, M. Wilkinson, L. R. Marshall, E. C. Rosser, A. Radziszewska, H. Peckham, C. Ciurtin, L. R. Wedderburn, R. Beale, C. Swanton, S. Gandhi, B. Stockinger, J. McCauley, S. J. Gamblin, L. E. McCoy, P. Cherepanov, E. Nastouli, G. Kassiotis, Preexisting and de novo humoral immunity to SARS-CoV-2 in humans. *Science* **370**, 1339–1343 (2020). doi:10.1126/science.abe1107 Medline
30. P. Nguyen-Contant, A. K. Embong, P. Kanagaiah, F. A. Chaves, H. Yang, A. R. Branche, D. J. Topham, M. Y. Sangster, S Protein-Reactive IgG and Memory B Cell Production after Human SARS-CoV-2 Infection Includes Broad Reactivity to the S2 Subunit. *mBio* **11**, e01991-20 (2020). doi:10.1128/mBio.01991-20 Medline
31. M. R. Filbin, A. Mehta, A. M. Schneider, K. R. Kays, J. R. Guess, M. Gentili, B. G. Fenyves, N. C. Charland, A. L. K. Gonye, I. Gushterova, H. K. Khanna, T. J. LaSalle, K. M. Lavin-Parsons, B. M. Lilley, C. L. Lodenstein, K. Manakongtreecheep, J. D. Margolin, B. N. McKaig, M. Rojas-Lopez, B. C. Russo, N. Sharma, J. Tantivit, M. F. Thomas, R. E. Gerszten, G. S. Heimberg, P. J. Hoover, D. J. Lieb, B. Lin, D. Ngo, K. Pelka, M. Reyes, C. S. Smillie, A. Waghray, T. E. Wood, A. S. Zajac, L. L. Jennings, I. Grundberg, R. P. Bhattacharyya, B. A. Parry, A.-C. Villani, M. Sade-Feldman, N. Hacohen, M. B. Goldberg, Longitudinal proteomic analysis of severe COVID-19 reveals survival-associated signatures, tissue-specific cell death, and cell-cell interactions. *Cell Rep Med.* **2**, 100287 (2021). doi:10.1016/j.xcrm.2021.100287 Medline
32. K. K. Yu, S. Fischinger, M. T. Smith, C. Atyeo, D. Cizmeci, C. R. Wolf, E. D. Layton, J. K. Logue, M. S. Aguilar, K. Shuey, C. Loos, J. Yu, N. Franko, R. Y. Choi, A. Wald, D. H. Barouch, D. M. Koelle, D. Lauffenburger, H. Y. Chu, G. Alter, C. Seshadri, Comorbid illnesses are associated with altered adaptive immune responses to SARS-CoV-2. *JCI Insight* **6**, e146242 (2021). doi:10.1172/jci.insight.146242 Medline
33. N. Kaneko, H.-H. Kuo, J. Boucau, J. R. Farmer, H. Allard-Chamard, V. S. Mahajan, A. Piechocka-Trocha, K. Lefteri, M. Osborn, J. Bals, Y. C. Bartsch, N. Bonheur, T. M. Caradonna, J. Chevalier, F. Chowdhury, T. J. Diefenbach, K. Einkauf, J. Fallon, J. Feldman, K. K. Finn, P. Garcia-Broncano, C. A. Hartana, B. M. Hauser, C. Jiang, P. Kaplonek, M. Karpell, E. C. Koscher, X. Lian, H. Liu, J. Liu, N. L. Ly, A. R. Michell, Y. Rassadkina, K. Seiger, L. Sessa, S. Shin, N. Singh, W. Sun, X. Sun, H. J. Ticheli, M. T. Waring, A. L. Zhu, G. Alter, J. Z. Li, D. Lingwood, A. G. Schmidt, M. Lichterfeld, B. D. Walker, X. G. Yu, R. F. Padera Jr., S. Pillai; Massachusetts Consortium on Pathogen Readiness Specimen Working Group, Loss of Bcl-6-Expressing T Follicular Helper Cells and Germinal Centers in COVID-19. *Cell* **183**, 143–157.e13 (2020). doi:10.1016/j.cell.2020.08.025 Medline
34. WHO. WHO R&D Blueprint - Novel Coronavirus, COVID-19 Therapeutic Trial Synopsis. 2020.
35. A. Gasmi, M. Peana, L. Pivina, S. Srinath, A. Gasmi Benahmed, Y. Semenova, A. Menzel, M. Dadar, G. Bjørklund, Interrelations between COVID-19 and other disorders. *Clin. Immunol.* **224**, 108651 (2021). doi:10.1016/j.clim.2020.108651 Medline
36. M. Huang, Q.-B. Lu, H. Zhao, Y. Zhang, Z. Sui, L. Fang, D. Liu, X. Sun, K. Peng, W. Liu, W. Guan, Temporal antibody responses to SARS-CoV-2 in patients of coronavirus disease 2019. *Cell Discov.* **6**, 64 (2020). doi:10.1038/s41421-020-00209-2 Medline
37. L. Premkumar, B. Segovia-Chumbez, R. Jadi, D. R. Martinez, R. Raut, A. Markmann, C. Cornaby, L. Bartelt, S. Weiss, Y. Park, C. E. Edwards, E. Weimer, E. M. Scherer, N. Rouphael, S. Edupuganti, D. Weiskopf, L. V. Tse, Y. J. Hou, D. Margolis, A. Sette, M. H. Collins, J. Schmitz, R. S. Baric, A. M. de Silva, The receptor binding domain of the viral spike protein is an immunodominant and highly specific target of antibodies in SARS-CoV-2 patients. *Sci. Immunol.* **5**, eabc8413 (2020). doi:10.1126/sciimmunol.abc8413 Medline
38. B. Isho, K. T. Abe, M. Zuo, A. J. Jamal, B. Rathod, J. H. Wang, Z. Li, G. Chao, O. L. Rojas, Y. M. Bang, A. Pu, N. Christie-Holmes, C. Gervais, D. Ceccarelli, P. Samavarchi-Tehrani, F. Guvenc, P. Budylowski, A. Li, A. Paterson, F. Y. Yue, L. M. Marin, L. Caldwell, J. L. Wrana, K. Colwill, F. Sicheri, S. Mubareka, S. D. Gray-Owen, S. J. Drews, W. L. Siqueira, M. Barrios-Rodiles, M. Ostrowski, J. M. Rini, Y. Durocher, A. J. McGeer, J. L. Gommerman, A.-C. Gingras, Persistence of serum and saliva antibody responses to SARS-CoV-2 spike antigens in COVID-19 patients. *Sci. Immunol.* **5**, eabe5511 (2020). doi:10.1126/sciimmunol.abe5511 Medline
39. F. Pinotti, P. S. Wikramaratna, U. Obolski, R. S. Paton, D. S. C. Damineli, L. C. J. Alcantara, M. Giovanetti, S. Gupta, J. Lourenço, Potential impact of individual exposure histories to endemic human coronaviruses on age-dependent severity of COVID-19. *BMC Med.* **19**, 19 (2021). doi:10.1186/s12916-020-01887-1 Medline
40. D. Aran, D. C. Beachler, S. Lanes, J. M. Overhage, Prior presumed coronavirus infection reduces COVID-19 risk: A cohort study. *J. Infect.* **81**, 923–930 (2020). doi:10.1016/j.jinf.2020.10.023 Medline
41. W. Fierz, B. Walz, Antibody Dependent Enhancement Due to Original Antigenic Sin and the Development of SARS. *Front. Immunol.* **11**, 1120 (2020). doi:10.3389/fimmu.2020.01120 Medline
42. K. W. Ng, N. Faulkner, G. H. Cornish, A. Rosa, R. Harvey, S. Hussain, R. Ulferts, C. Earl, A. G. Wrobel, D. J. Benton, C. Roustan, W. Bolland, R. Thompson, A. Agua-Doce, P. Hobson, J. Heaney, H. Rickman, S. Paraskevopoulou, C. F. Houlihan, K. Thomson, E. Sanchez, G. Y. Shin, M. J. Spyer, D. Joshi, N. O'Reilly, P. A. Walker, S. Kjaer, A. Riddell, C. Moore, B. R. Jebson, M. Wilkinson, L. R. Marshall, E. C. Rosser, A. Radziszewska, H. Peckham, C. Ciurtin, L. R. Wedderburn, R. Beale, C. Swanton, S. Gandhi, B. Stockinger, J. McCauley, S. J. Gamblin, L. E. McCoy, P. Cherepanov, E. Nastouli, G. Kassiotis, Preexisting and de novo humoral immunity to SARS-CoV-2 in humans. *Science* **370**, 1339–1343 (2020). doi:10.1126/science.abe1107 Medline
43. A. Sokal, P. Chappert, G. Barba-Spaeth, A. Roeser, S. Fourati, I. Azzaoui, A. Vandenberghe, I. Fernandez, A. Meola, M. Bouvier-Alias, E. Crickx, A. Beldi-Ferchiou, S. Hue, L. Languille, M. Michel, S. Baloul, F. Noizat-Pirenne, M. Luka, J. Mégret, M. Ménager, J.-M. Pawlotsky, S. Fillatreau, F. A. Rey, J.-C. Weill, C.-A. Reynaud, M. Mahévas, Maturation and persistence of the anti-SARS-CoV-2 memory B cell response. *Cell* **184**, 1201–1213.e14 (2021). doi:10.1016/j.cell.2021.01.050 Medline
44. U. Greenbaum *et al*., High levels of common cold coronavirus antibodies in convalescent plasma are associated with improved survival in COVID-19 patients.

medRxiv. 2021.
45. L. Guo, Y. Wang, L. Kang, Y. Hu, L. Wang, J. Zhong, H. Chen, L. Ren, X. Gu, G. Wang, C. Wang, X. Dong, C. Wu, L. Han, Y. Wang, G. Fan, X. Zou, H. Li, J. Xu, Q. Jin, B. Cao, J. Wang, Cross-reactive antibody against human coronavirus OC43 spike protein correlates with disease severity in COVID-19 patients: A retrospective study. *Emerg. Microbes Infect.* **10**, 664–676 (2021). doi:10.1080/22221751.2021.1905488 Medline
46. B. M. Westerhuis *et al*., Severe COVID-19 patients display a back boost of seasonal coronavirus-specific antibodies. medRxiv. 2020:2020.10.10.20210070.
47. M. E. Killerby, H. M. Biggs, A. Haynes, R. M. Dahl, D. Mustaquim, S. I. Gerber, J. T. Watson, Human coronavirus circulation in the United States 2014-2017. *J. Clin. Virol.* **101**, 52–56 (2018). doi:10.1016/j.jcv.2018.01.019 Medline
48. M. Throsby, E. van den Brink, M. Jongeneelen, L. L. M. Poon, P. Alard, L. Cornelissen, A. Bakker, F. Cox, E. van Deventer, Y. Guan, J. Cinatl, J. ter Meulen, I. Lasters, R. Carsetti, M. Peiris, J. de Kruif, J. Goudsmit, Heterosubtypic neutralizing monoclonal antibodies cross-protective against H5N1 and H1N1 recovered from human IgM+ memory B cells. *PLOS ONE* **3**, e3942 (2008). doi:10.1371/journal.pone.0003942 Medline
49. C. Bohannon, R. Powers, L. Satyabhama, A. Cui, C. Tipton, M. Michaeli, I. Skountzou, R. S. Mittler, S. H. Kleinstein, R. Mehr, F. E.-Y. Lee, I. Sanz, J. Jacob, Long-lived antigen-induced IgM plasma cells demonstrate somatic mutations and contribute to long-term protection. *Nat. Commun.* **7**, 11826 (2016). doi:10.1038/ncomms11826 Medline
50. Y. C. Bartsch, S. Fischinger, S. M. Siddiqui, Z. Chen, J. Yu, M. Gebre, C. Atyeo, M. J. Gorman, A. L. Zhu, J. Kang, J. S. Burke, M. Slein, M. J. Gluck, S. Beger, Y. Hu, J. Rhee, E. Petersen, B. Mormann, M. S. Aubin, M. A. Hasdianda, G. Jambaulikar, E. W. Boyer, P. C. Sabeti, D. H. Barouch, B. D. Julg, E. R. Musk, A. S. Menon, D. A. Lauffenburger, E. J. Nilles, G. Alter, Discrete SARS-CoV-2 antibody titers track with functional humoral stability. *Nat. Commun.* **12**, 1018 (2021). doi:10.1038/s41467-021-21336-8 Medline
51. E. Dong, H. Du, L. Gardner, An interactive web-based dashboard to track COVID-19 in real time. *Lancet Infect. Dis.* **20**, 533–534 (2020). doi:10.1016/S1473-3099(20)30120-1 Medline
52. WHO. Coronavirus Disease (COVID-19) Situation Dashboard (WHO, accessed 18 Jan 2021). 2021.
53. K. Sun, W. Wang, L. Gao, Y. Wang, K. Luo, L. Ren, Z. Zhan, X. Chen, S. Zhao, Y. Huang, Q. Sun, Z. Liu, M. Litvinova, A. Vespignani, M. Ajelli, C. Viboud, H. Yu, Transmission heterogeneities, kinetics, and controllability of SARS-CoV-2. *Science* **371**, eabe2424 (2021). doi:10.1126/science.abe2424 Medline
54. D. K. Chu, E. A. Akl, S. Duda, K. Solo, S. Yaacoub, H. J. Schünemann; COVID-19 Systematic Urgent Review Group Effort (SURGE) study authors, Physical distancing, face masks, and eye protection to prevent person-to-person transmission of SARS-CoV-2 and COVID-19: A systematic review and meta-analysis. *Lancet* **395**, 1973–1987 (2020). doi:10.1016/S0140-6736(20)31142-9 Medline
55. H. Li, C. Chen, F. Hu, J. Wang, Q. Zhao, R. P. Gale, Y. Liang, Impact of corticosteroid therapy on outcomes of persons with SARS-CoV-2, SARS-CoV, or MERS-CoV infection: A systematic review and meta-analysis. *Leukemia* **34**, 1503–1511 (2020). doi:10.1038/s41375-020-0848-3 Medline
56. O. S. Adeniji, L. B. Giron, M. Purwar, N. F. Zilberstein, A. J. Kulkarni, M. W. Shaikh, R. A. Balk, J. N. Moy, C. B. Forsyth, Q. Liu, H. Dweep, A. Kossenkov, D. B. Weiner, A. Keshavarzian, A. Landay, M. Abdel-Mohsen, COVID-19 Severity Is Associated with Differential Antibody Fc-Mediated Innate Immune Functions. *mBio* **12**, e00281–e21 (2021). Medline
57. O. S. Adeniji *et al*., COVID-19 Severity Is Associated with Differential Antibody Fc-Mediated Innate Immune Functions. *mBio* **12**, (2021).
58. W. S. Lee, K. J. Selva, S. K. Davis, B. D. Wines, A. Reynaldi, R. Esterbauer, H. G. Kelly, E. R. Haycroft, H.-X. Tan, J. A. Juno, A. K. Wheatley, P. M. Hogarth, D. Cromer, M. P. Davenport, A. W. Chung, S. J. Kent, Decay of Fc-dependent antibody functions after mild to moderate COVID-19. *Cell Rep. Med.* **2**, 100296 (2021). doi:10.1016/j.xcrm.2021.100296 Medline
59. S. P. Anand, J. Prévost, M. Nayrac, G. Beaudoin-Bussières, M. Benlarbi, R. Gasser, N. Brassard, A. Laumaea, S. Y. Gong, C. Bourassa, E. Brunet-Ratnasingham, H. Medjahed, G. Gendron-Lepage, G. Goyette, L. Gokool, C. Morrisseau, P. Bégin, V. Martel-Laferrière, C. Tremblay, J. Richard, R. Bazin, R. Duerr, D. E. Kaufmann, A. Finzi, Longitudinal analysis of humoral immunity against SARS-CoV-2 Spike in convalescent individuals up to 8 months post-symptom onset. *Cell Rep Med.* **2**, 100290 (2021). doi:10.1016/j.xcrm.2021.100290 Medline
60. J. Dufloo, L. Grzelak, I. Staropoli, Y. Madec, L. Tondeur, F. Anna, S. Pelleau, A. Wiedemann, C. Planchais, J. Buchrieser, R. Robinot, M.-N. Ungeheuer, H. Mouquet, P. Charneau, M. White, Y. Lévy, B. Hoen, A. Fontanet, O. Schwartz, T. Bruel, Asymptomatic and symptomatic SARS-CoV-2 infections elicit polyfunctional antibodies. *Cell Rep Med.* **2**, 100275 (2021). doi:10.1016/j.xcrm.2021.100275 Medline
61. K. E. Stephenson, M. Le Gars, J. Sadoff, A. M. de Groot, D. Heerwegh, C. Truyers, C. Atyeo, C. Loos, A. Chandrashekar, K. McMahan, L. H. Tostanoski, J. Yu, M. S. Gebre, C. Jacob-Dolan, Z. Li, S. Patel, L. Peter, J. Liu, E. N. Borducchi, J. P. Nkolola, M. Souza, C. S. Tan, R. Zash, B. Julg, R. R. Nathavitharana, R. L. Shapiro, A. A. Azim, C. D. Alonso, K. Jaegle, J. L. Ansel, D. G. Kanjilal, C. J. Guiney, C. Bradshaw, A. Tyler, T. Makoni, K. E. Yanosick, M. S. Seaman, D. A. Lauffenburger, G. Alter, F. Struyf, M. Douoguih, J. Van Hoof, H. Schuitemaker, D. H. Barouch, Immunogenicity of the Ad26.COV2.S Vaccine for COVID-19. *JAMA* **325**, 1535–1544 (2021). doi:10.1001/jama.2021.3645 Medline
62. A. Tauzin *et al*., A single BNT162b2 mRNA dose elicits antibodies with Fc-mediated effector functions and boost pre-existing humoral and T cell responses. bioRxiv. 2021:2021.03.18.435972.
63. J. Ravetch *et al*., Fc-engineered antibody therapeutics with improved efficacy against COVID-19. Res Sq. 2021.
64. C. E. Z. Chan *et al*., The Fc-mediated effector functions of a potent SARS-CoV-2 neutralizing antibody, SC31, isolated from an early convalescent COVID-19 patient, are essential for the optimal therapeutic efficacy of the antibody. bioRxiv. 2020:2020.10.26.355107.
65. C. G. Rappazzo, L. V. Tse, C. I. Kaku, D. Wrapp, M. Sakharkar, D. Huang, L. M. Deveau, T. J. Yockachonis, A. S. Herbert, M. B. Battles, C. M. O'Brien, M. E. Brown, J. C. Geoghegan, J. Belk, L. Peng, L. Yang, Y. Hou, T. D. Scobey, D. R. Burton, D. Nemazee, J. M. Dye, J. E. Voss, B. M. Gunn, J. S. McLellan, R. S. Baric, L. E. Gralinski, L. M. Walker, Broad and potent activity against SARS-like viruses by an engineered human monoclonal antibody. *Science* **371**, 823–829 (2021). doi:10.1126/science.abf4830 Medline
66. E. S. Winkler, P. Gilchuk, J. Yu, A. L. Bailey, R. E. Chen, Z. Chong, S. J. Zost, H. Jang, Y. Huang, J. D. Allen, J. B. Case, R. E. Sutton, R. H. Carnahan, T. L. Darling, A. C. M. Boon, M. Mack, R. D. Head, T. M. Ross, J. E. Crowe Jr., M. S. Diamond, Human neutralizing antibodies against SARS-CoV-2 require intact Fc effector functions for optimal therapeutic protection. *Cell* **184**, 1804–1820.e16 (2021). doi:10.1016/j.cell.2021.02.026 Medline
67. P. Zimmermann, N. Curtis, Why is COVID-19 less severe in children? A review of the proposed mechanisms underlying the age-related difference in severity of SARS-CoV-2 infections. *Arch. Dis. Child.* **106**, 429–439 (2020). doi:10.1136/archdischild-2020-320338 Medline
68. Z. Yi, Y. Ling, X. Zhang, J. Chen, K. Hu, Y. Wang, W. Song, T. Ying, R. Zhang, H. Lu, Z. Yuan, Functional mapping of B-cell linear epitopes of SARS-CoV-2 in COVID-19 convalescent population. *Emerg. Microbes Infect.* **9**, 1988–1996 (2020). doi:10.1080/22221751.2020.1815591 Medline
69. D. Pinto *et al*., A human antibody that broadly neutralizes betacoronaviruses protects against SARS-CoV-2 by blocking the fusion machinery. bioRxiv. 2021:2021.05.09.442808.
70. G. Song, W. T. He, S. Callaghan, F. Anzanello, D. Huang, J. Ricketts, J. L. Torres, N. Beutler, L. Peng, S. Vargas, J. Cassell, M. Parren, L. Yang, C. Ignacio, D. M. Smith, J. E. Voss, D. Nemazee, A. B. Ward, T. Rogers, D. R. Burton, R. Andrabi, Cross-reactive serum and memory B-cell responses to spike protein in SARS-CoV-2 and endemic coronavirus infection. *Nat. Commun.* **12**, 2938 (2021). doi:10.1038/s41467-021-23074-3 Medline
71. T. A. Von Holle, M. A. Moody, Influenza and Antibody-Dependent Cellular Cytotoxicity. *Front. Immunol.* **10**, 1457 (2019). doi:10.3389/fimmu.2019.01457 Medline
72. E. S. Rosenberg, D. R. Holtgrave, V. Dorabawila, M. Conroy, D. Greene, E. Lutterloh, B. Backenson, D. Hoefer, J. Morne, U. Bauer, H. A. Zucker, New COVID-19 Cases and Hospitalizations Among Adults, by Vaccination Status - New York, May 3-July 25, 2021. *MMWR Morb. Mortal. Wkly. Rep.* **70**, 1150–1155 (2021). doi:10.15585/mmwr.mm7034e1 Medline

73. D. M. Weinreich, S. Sivapalasingam, T. Norton, S. Ali, H. Gao, R. Bhore, B. J. Musser, Y. Soo, D. Rofail, J. Im, C. Perry, C. Pan, R. Hosain, A. Mahmood, J. D. Davis, K. C. Turner, A. T. Hooper, J. D. Hamilton, A. Baum, C. A. Kyratsous, Y. Kim, A. Cook, W. Kampman, A. Kohli, Y. Sachdeva, X. Graber, B. Kowal, T. DiCioccio, N. Stahl, L. Lipsich, N. Braunstein, G. Herman, G. D. Yancopoulos; Trial Investigators, REGN-COV2, a Neutralizing Antibody Cocktail, in Outpatients with Covid-19. *N. Engl. J. Med.* **384**, 238–251 (2021). doi:10.1056/NEJMoa2035002 Medline
74. I. Skountzou, L. Satyabhama, A. Stavropoulou, Z. Ashraf, E. S. Esser, E. Vassilieva, D. Koutsonanos, R. Compans, J. Jacob, Influenza virus-specific neutralizing IgM antibodies persist for a lifetime. *Clin. Vaccine Immunol.* **21**, 1481–1489 (2014). doi:10.1128/CVI.00374-14 Medline
75. M. Auladell, X. Jia, L. Hensen, B. Chua, A. Fox, T. H. O. Nguyen, P. C. Doherty, K. Kedzierska, Recalling the Future: Immunological Memory Toward Unpredictable Influenza Viruses. *Front. Immunol.* **10**, 1400 (2019). doi:10.3389/fimmu.2019.01400 Medline
76. A. C. Davis, K. H. Roux, M. J. Shulman, On the structure of polymeric IgM. *Eur. J. Immunol.* **18**, 1001–1008 (1988). doi:10.1002/eji.1830180705 Medline
77. T. D. Randall, L. B. King, R. B. Corley, The biological effects of IgM hexamer formation. *Eur. J. Immunol.* **20**, 1971–1979 (1990). doi:10.1002/eji.1830200915 Medline
78. M. R. Ehrenstein, C. A. Notley, The importance of natural IgM: Scavenger, protector and regulator. *Nat. Rev. Immunol.* **10**, 778–786 (2010). doi:10.1038/nri2849 Medline
79. J. M. Dan, J. Mateus, Y. Kato, K. M. Hastie, E. D. Yu, C. E. Faliti, A. Grifoni, S. I. Ramirez, S. Haupt, A. Frazier, C. Nakao, V. Rayaprolu, S. A. Rawlings, B. Peters, F. Krammer, V. Simon, E. O. Saphire, D. M. Smith, D. Weiskopf, A. Sette, S. Crotty, Immunological memory to SARS-CoV-2 assessed for up to 8 months after infection. *Science* **371**, eabf4063 (2021). doi:10.1126/science.abf4063 Medline
80. G. E. Hartley, E. S. J. Edwards, P. M. Aui, N. Varese, S. Stojanovic, J. McMahon, A. Y. Peleg, I. Boo, H. E. Drummer, P. M. Hogarth, R. E. O'Hehir, M. C. van Zelm, Rapid generation of durable B cell memory to SARS-CoV-2 spike and nucleocapsid proteins in COVID-19 and convalescence. *Sci. Immunol.* **5**, eabf8891 (2020). doi:10.1126/sciimmunol.abf8891 Medline
81. K. L. Newell, D. C. Clemmer, J. B. Cox, Y. I. Kayode, V. Zoccoli-Rodriguez, H. E. Taylor, T. P. Endy, J. R. Wilmore, G. M. Winslow, Switched and unswitched memory B cells detected during SARS-CoV-2 convalescence correlate with limited symptom duration. *PLOS ONE* **16**, e0244855 (2021). doi:10.1371/journal.pone.0244855 Medline
82. J. H. Kim, I. Skountzou, R. Compans, J. Jacob, Original antigenic sin responses to influenza viruses. *J. Immunol.* **183**, 3294–3301 (2009). doi:10.4049/jimmunol.0900398 Medline
83. E. P. Brown, K. G. Dowell, A. W. Boesch, E. Normandin, A. E. Mahan, T. Chu, D. H. Barouch, C. Bailey-Kellogg, G. Alter, M. E. Ackerman, Multiplexed Fc array for evaluation of antigen-specific antibody effector profiles. *J. Immunol. Methods* **443**, 33–44 (2017). doi:10.1016/j.jim.2017.01.010 Medline

Acknowledgments: We thank Nancy Zimmerman, Mark and Lisa Schwartz, an anonymous donor (financial support), Terry and Susan Ragon, and the SAMANA Kay MGH Research Scholars award for their support. **Funding:** We acknowledge support from the Ragon Institute of MGH, MIT and Harvard, the Massachusetts Consortium on Pathogen Readiness (MassCPR), the NIH (3R37AI080289-11S1, R01AI146785, U19AI42790-01, U19AI135995-02, U19AI42790-01, 1U01CA260476 – 01, CIVIC75N93019C00052), the Gates Foundation Global Health Vaccine Accelerator Platform funding (OPP1146996 and INV-001650), and the Musk Foundation. MBG, MRF, and NH received support from the American Lung Association and the MGH Executive Committee on Research. This work was also supported by the Translational Research Institute for Space Health through NASA Cooperative Agreement NNX16AO69A **Author Contributions:** P.K., C.W., D.A.L., and G.A. analyzed and interpreted the data. P.K., S.F., Y.C.B., M.J.G., K.B., and J.K. performed experiments. C.W. and D.A.L performed the analysis. M.G., M.F., N.H., D.D., B.C.R., M.R.L., K.M., J.T., and M.F.T managed samples and data collection for acutely ill COVID-19 patients. B.M.L., C.L.L., B.N.M., H.K.K., and J.D,M, collected samples for acutely ill COVID-19 patient. A.L.K.G., I.G., T.J.L., M.S.F., and N.S. processed samples for acutely ill COVID-19 patient. P.M., A.S.M., E.J.N. and E.R.M. managed samples and data collection for the Community-acquired COVID-19 cohort. R.M., C.H., J.F., A.S., and J.S.M., E.S.F. produced SARS-CoV-2 and OC43 antigens. G.A. and D.A.L. supervised the project. P.K., G.A., and C.W. drafted the manuscript. All authors critically reviewed the manuscript. **Competing of interests:** G.A. is a founder and equity holder for Seromyx Systems Inc., an employee and equity holder for Leyden Labs, has received financial support from Abbvie, BioNtech, GSK, Gilead, Merck, Moderna, Novartis, Pfizer, and Sanofi, and has a patent pending with systems serology. D.D., P.M., A.S.M, and E.R.M. are employees of Space Exploration Technologies Corp. E.S.F. is a founder, scientific advisor and equity holder for: Jengu Therapeutics (board member), Neomorph Inc and Civetta Therapeutics; an equity holder in C4 Therapeutics (CCCC); and a consultant to Novartis, Sanofi, AbbVie, Pfizer, Astellas, EcoR1 capital and Deerfield. The Fischer lab receives or has received research funding from Novartis, Ajax, and Astellas not related to this work. All other authors have declared that no conflict of interest exists. **Data and materials availability:** Codes code and scripts used for this study have been deposited in a public database and can be found here: https://sandbox.zenodo.org/record/905676#.YSkWQI5KhPZ. All data needed to evaluate the conclusions in the paper are also available in the dataset folder https://sandbox.zenodo.org/record/905676#.YSkWQI5KhPZ. All data needed to evaluate the conclusions in the paper are present in the paper or the Supplementary Materials. This work is licensed under a Creative Commons Attribution 4.0 International (CC BY 4.0) license, which permits unrestricted use, distribution, and reproduction in any medium, provided the original work is properly cited. To view a copy of this license, visit https://creativecommons.org/licenses/by/4.0/. This license does not apply to figures/photos/artwork or other content included in the article that is credited to a third party; obtain authorization from the rights holder before using such material.

Submitted 5 May 2021
Accepted 1 September 2021
Published First Release 9 September 2021
10.1126/sciimmunol.abj2901

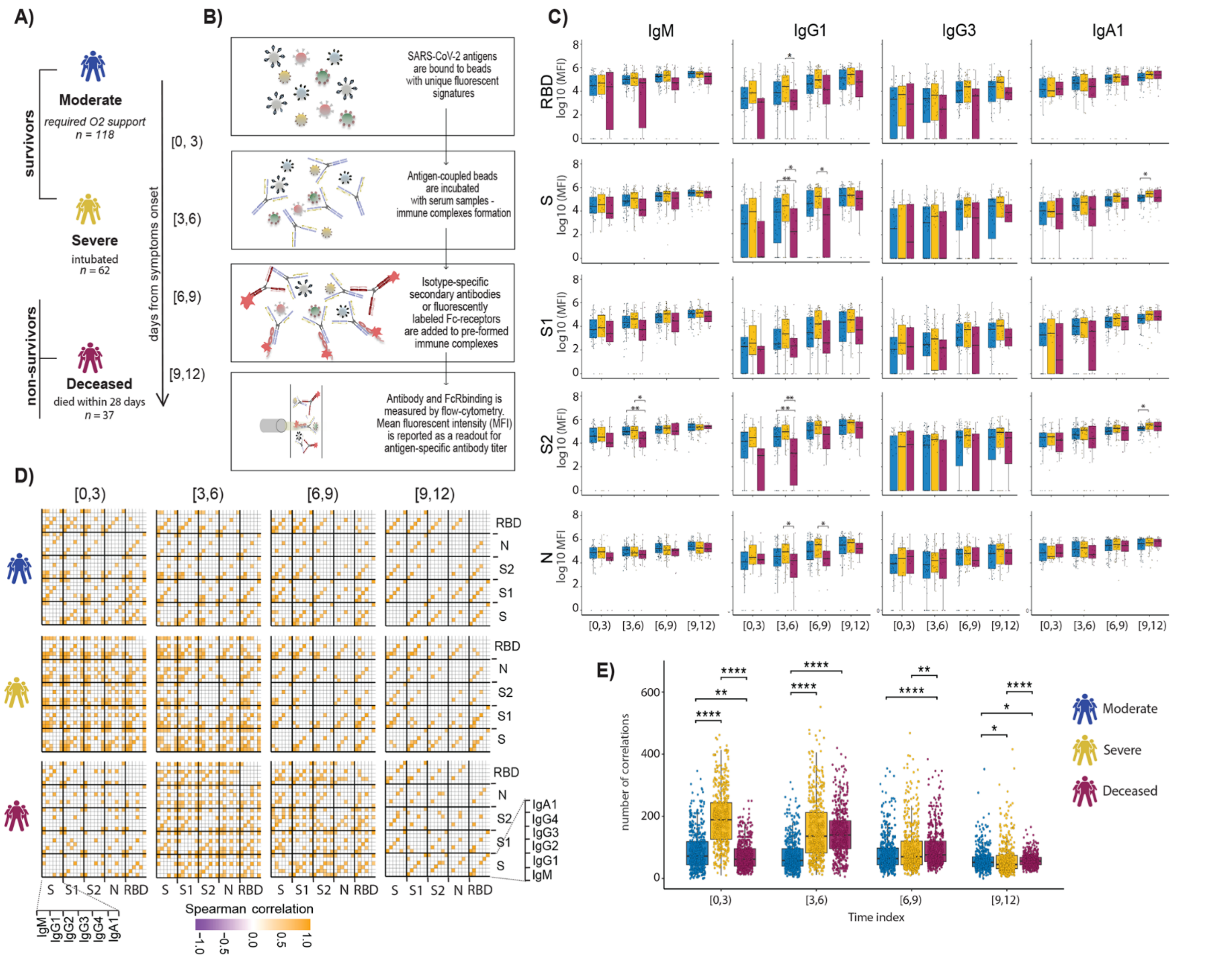

Fig. 1. Evolution of early SARS-CoV-2 specific humoral immune responses following symptom onset across acutely ill COVID-19 patients. (A) The cartoon shows the study groups based on COVID-19 severity: 217 COVID-19-infected patients were sampled on days 0, 3, and 7 after admission to the hospital. Patients were classified into three groups based on the maximal acuity within 28 days of enrollment: Moderate in blue: hospitalized that required supplemental oxygen (*n* = 118). Severe in yellow: intubation, mechanical ventilation, and survival to 28 days (*n* = 62). Deceased in purple: death within 28 days (*n* = 37). Based on the day of symptom onset, the samples were divided into four temporal groups: [0, 3), [3, 6), [6, 9), [9, 12). **(B)** A graphical summary of the Luminex assay **(C)** The whisker plots show the distribution of antibody titers across moderate (blue), severe (yellow), and deceased (purple) over the study time course. The solid black line represents the median, and the box boundary (upper and below) represents the first and third quartiles. The dots show the scaled values of each sample. A two-sample Wilcox test was used to evaluate statistical differences across groups for all the intervals and features. The P-values were corrected from multiple hypothesis testing using the Benjamini-Hochbery procedure per each interval. Significance corresponds to adjusted P-values. (* $p < 0.05$, ** $p < 0.01$). **(D)** The correlation heatmap shows pairwise Spearman correlation matrices of SARS-CoV-2-specific antibody response across COVID-19 severity groups (moderate, severe, and deceased) for all four intervals. Correlation coefficients are shown only if they are larger than 0.6 and statistically significant after Benjamini-Hochberg correction for multiple hypothesis testing. Negative correlations are indicated in purple, positive correlations are shown in orange. (**E**) The statistical evaluation of the effect of sample size. The spearman correlation is calculated by randomly selected ten samples per category for 500 runs. The number of statistically significant correlations (larger than 0.6) is calculated and tested by the Mann-Whitney U test. Significance corresponds to adjusted P-values. (* $p < 0.05$, ** $p < 0.01$, *** $p < 0.001$, **** $p < 0.0001$).

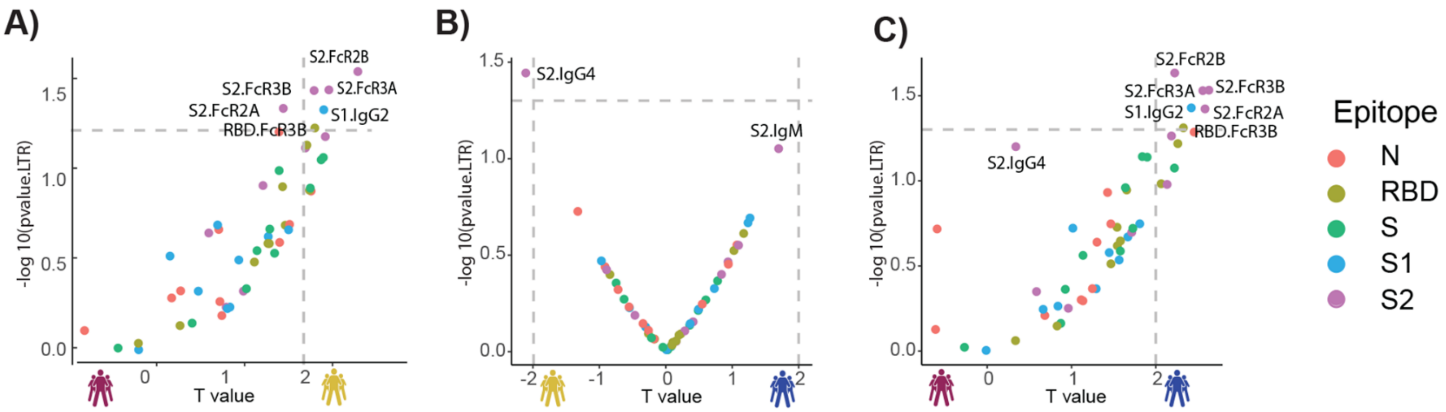

Fig. 2. Selective enrichment of S2-specific responses across COVID-19 patients. (A-C) Volcano plots of pairwise comparisons across pairs of each of the three groups highlight differences across groups controlling for age, BMI, heart, lung and kidney diseases. The volcano plots include comparisons of **(A)** individuals that passed away within 28 days (deceased) vs. severe survivors; **(B)** subjects who experienced moderate disease vs. severe survivors; **(C)** subjects who ultimately passed away (deceased) vs. subjects who developed moderate disease. The x-axis represents the *t* value of the full model, and the y-axis denotes the *p* values by likelihood ratio test comparing the null model and full model. The null/full model represents the association between each individual measurement (response) and all collected clinical information with/without disease severity (see methods). The horizontal gray dashed line denotes the *p*-value equals 0.05, and the vertical gray dashed line denotes a manually selected threshold (*t* values = 2).

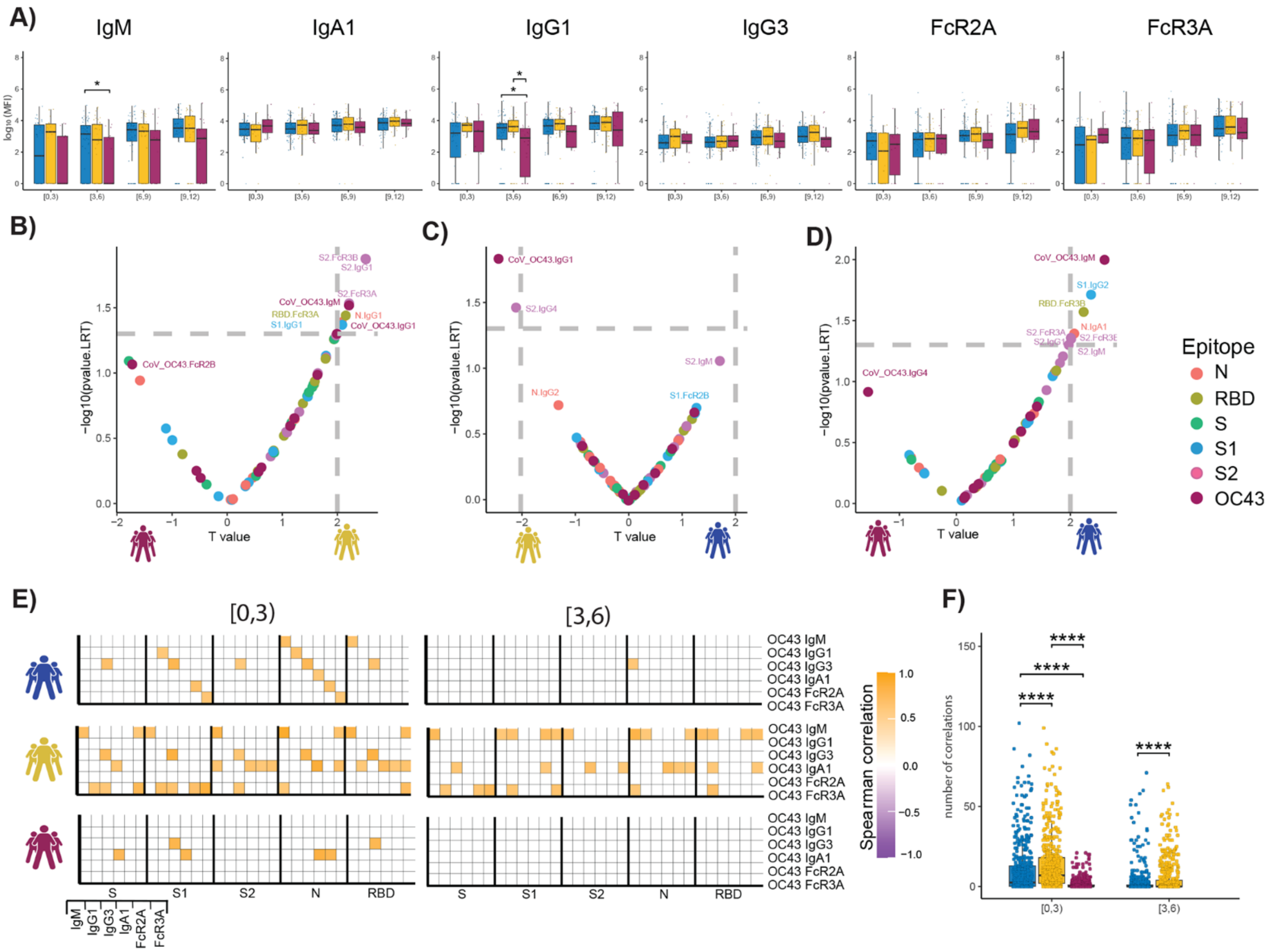

Fig. 3. The temporal evolution of the human OC43 specific humoral immune response. (A) The whisker bar graphs show the distribution of human OC43 receptor binding domain (RBD)-specific antibody titers and OC43-specific antibody mediated Fc-receptor binding profiles across moderate, severe, and non-survivor COVID-19 groups over the study time course. The solid black line represents the median and box boundary (upper and bottom). (B-D) The volcano plots show the pairwise comparisons across the three COVID-19 severity groups, (B) individuals that passed away within 28 days (deceased) vs. severe survivors; (C) subjects who experienced moderate disease vs. severe survivors; (D) subjects who ultimately passed away (deceased) vs. subjects who developed moderate disease, including human OC43 RBD-specific humoral immune data. (E) The correlation heatmap shows the pairwise Spearman correlation matrices between OC43-specific and SARS-CoV-2 antibody levels across three COVID-19 severity groups (moderate, severe, and non-survivors) across the study time course. The correlation coefficients were shown only if statistically significant (adjust *p*-value < 0.05) after Benjamini-Hochberg correction from multiple hypothesis testing. (F) The statistical evaluation of the effect of sample size. The spearman correlation is calculated by randomly selected ten samples per category for 500 runs (the deceased group in day interval [3,6), is not included since the number of samples is less than 10). The number of statistically significant correlations (larger than 0.6) is calculated and tested by the Mann-Whitney U test. Significance corresponds to adjusted P-values. (* p < 0.05, ** p < 0.01, *** p <0.001, **** p <0.0001).

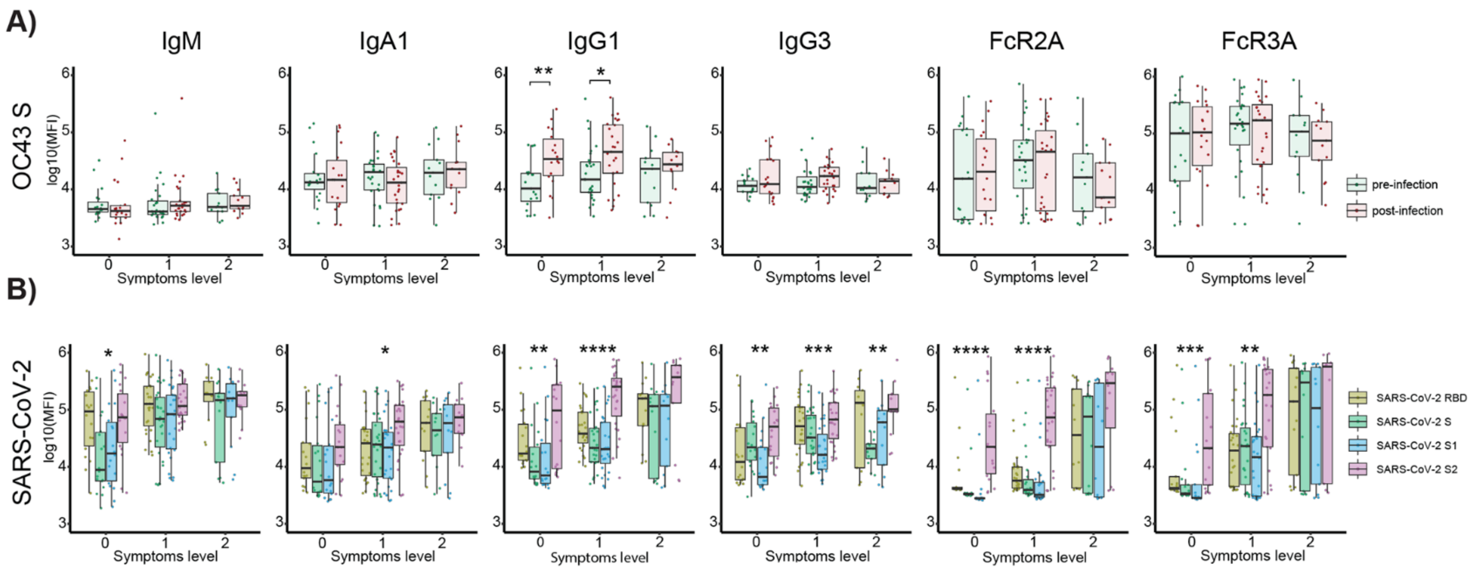

Fig. 4. SARS-CoV-2 S2-specific antibody functionality tracks with asymptomatic SARS-CoV-2 infection. **(A)** The whisker box plots show the overall humoral immune response to OC43 RBD-spike titers across a community based SARS-CoV-2 infection cohort divided by individuals that were asymptomatic (symptoms level 0) or experienced symptoms (symptoms level 1 or level 2, based on degree of symptoms) pre- and post-infection. **(B)** The bar graphs illustrate the SARS-CoV-2 specific humoral immune response across the RBD, S, S1, and S2 antigens across the same community based surveillance study divided by the degree of symptoms (symptoms levels). The dots show the scaled values of each sample. A two-sample Wilcox test was used to evaluate statistical differences across different epitopes for all the symptom categories. Significance corresponds to adjusted P-values. (* $p < 0.05$, ** $p < 0.01$, *** $p < 0.001$, **** $p < 0.0001$).
