# Supplementary material for: Early cross-coronavirus reactive signatures of humoral immunity against COVID-19
Source: Sci Immunol. 2021 Sep 9;6(64):eabj2901. doi: 10.1126/sciimmunol.abj2901 (PMC8943686; doi:10.1126/sciimmunol.abj2901)
Supplement: Supplementary file 2 — Figs. S1 to S4 Table S1 Legend for table S2 [file sciimmunol.abj2901_sm.pdf]

Science Immunology
AAAS

Supplementary Materials for

Early cross-coronavirus reactive signatures of humoral immunity against COVID-19

Paulina Kaplonek *et al.*

Corresponding author: Marcia B. Goldberg, marcia.goldberg@mgh.harvard.edu;
Michael R. Filbin, mfilbin@mgh.harvard.edu; Nir Hacohen, nhacohen@mgh.harvard.edu;
Douglas A. Lauffenburger, lauffen@mit.edu; Galit Alter, galter@partners.org

Sci. Immunol. **6**, eabj2901 (2021)
DOI: 10.1126/sciimmunol.abj2901

The PDF file includes:

Figs. S1 to S4
Table S1
Legend for table S2

Other Supplementary Material for this manuscript includes the following:

Table S2

Supplementary materials

Supplementary Figures and Tables:

Fig. S1. Distribution of RBD-, S-, S1-, S2- and N-specific antibody isotypes/ subclasses and their ability to bind Fc-receptors across acutely ill COVID-19 patients.

Fig. S2. The Spearman correlation between SARS-CoV-2 and human cCoV OC43 antibody levels and neutralization level over time.

Fig. S3. Temporal evolution of SARS-CoV-2 specific antibody.

Fig. S4. Defined symptom groups for community acquired-COVID-19 mild and asymptomatic individuals.

Table S1. Samples distribution across disease severity and days intervals since symptoms onset.

Table S2. The raw data (Excel datasheet).

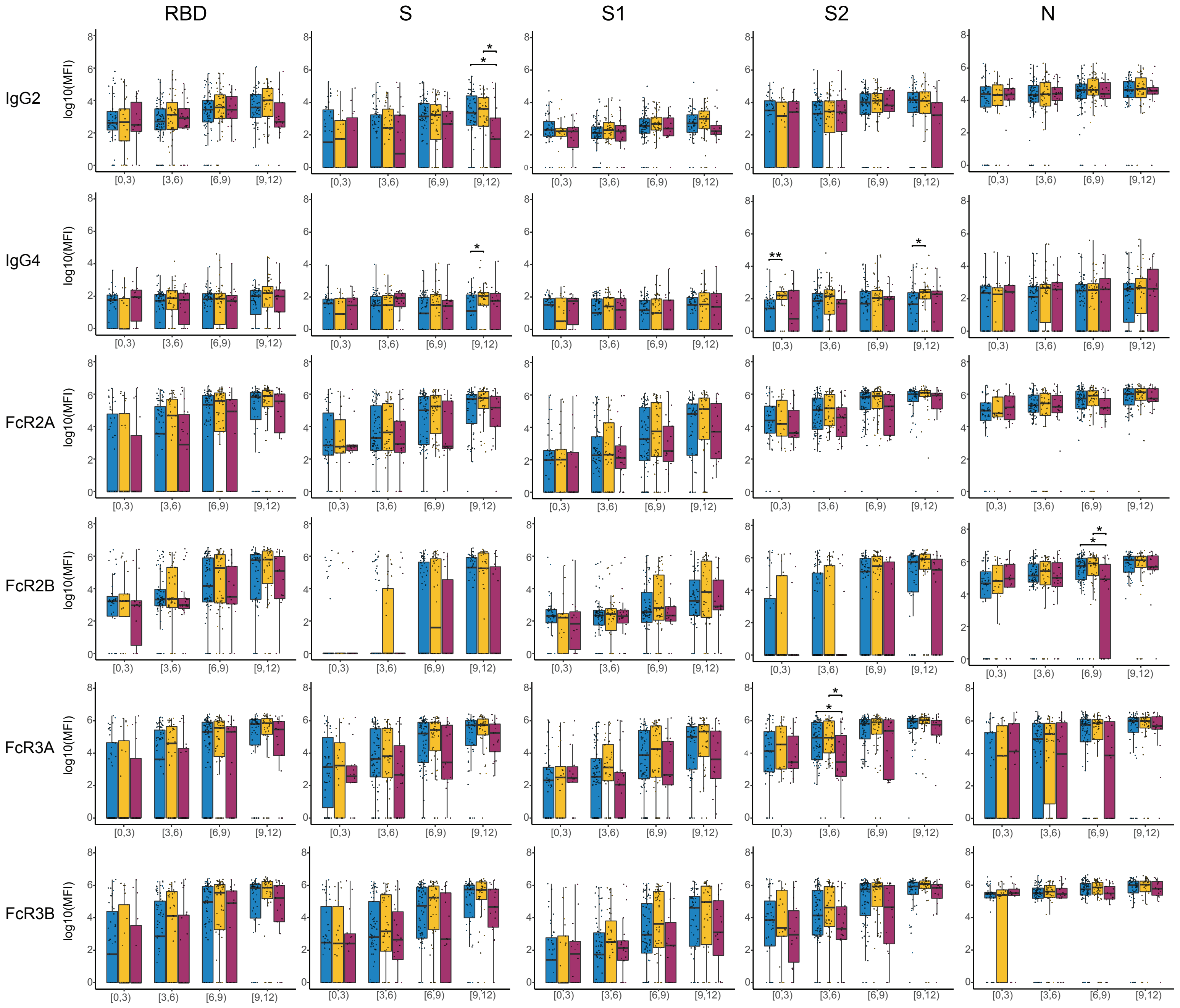

RBD
S
S1
S2
N
IgG2
IgG4
FcR2A
FcR2B
FcR3A
FcR3B
log10(MFI)
[0,3)
[3,6)
[6,9)
[9,12)

Fig. S1. Distribution of RBD-, S-, S1-, S2- and N-specific antibody isotypes/ subclasses and their ability to bind Fc-receptors across acutely ill COVID-19 patients.

The immune response of SARS-CoV-2 infected individuals was profiled against SARS-CoV-2 antigens. Distributions of IgG2, IgG4 as antibody titers and binding to FcγR2A, FcγR2B, FcγR3A, FcγR3B across moderate (blue), severe (yellow) and deceased (red) COVID-19 patients are shown as box-plots over the time intervals (0-3, 3-6, 6-9, 9-12) following symptom onset. The solid black line represents the median, and the upper and bottom lines of box plots show the first and third quartiles. Values are reported as log10 MFI. A two-sample Wilcox test was used to evaluate statistical differences across groups for all the intervals and features. The P-values were corrected from multiple hypothesis testing using the Benjamini-Hochberg procedure per each interval. Here, significance corresponds to the adjusted P-values. (* $p < 0.05$, ** $p < 0.01$).

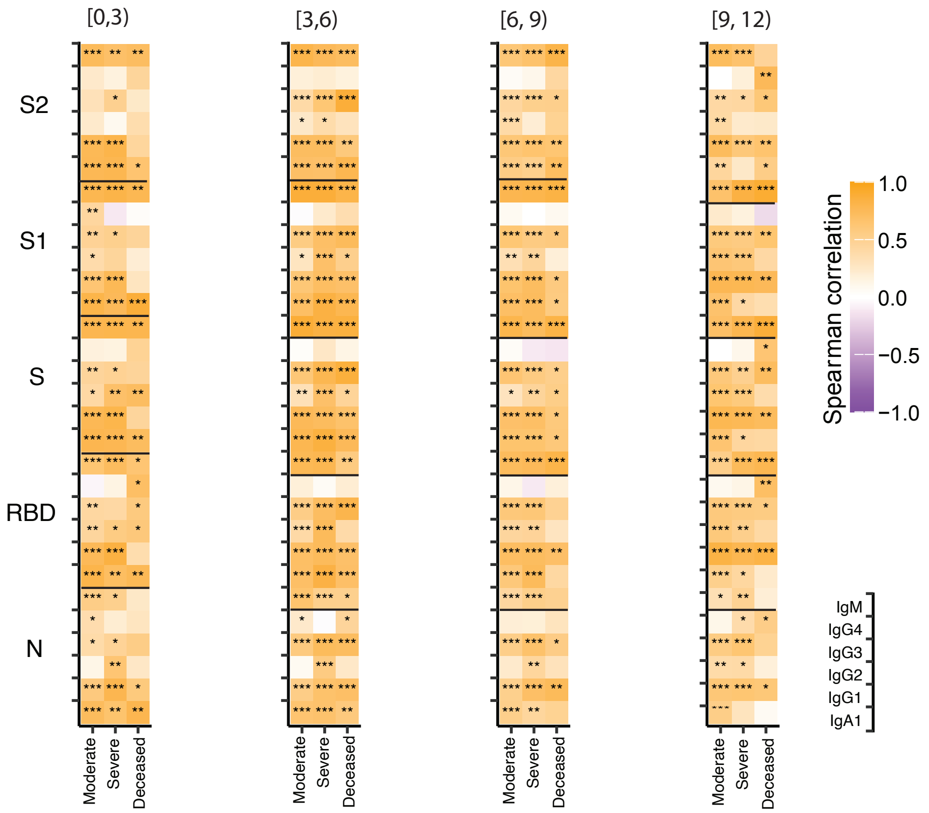
[0,3)
[3,6)
[6, 9)
[9, 12)
S2
S1
S
RBD
N
Moderate
Severe
Deceased
Spearman correlation
1.0
0.5
0.0
−0.5
−1.0
IgM
IgG4
IgG3
IgG2
IgG1
IgA1

Fig. S2. The Spearman correlation between SARS-CoV-2 and human cCoV OC43 antibody levels and neutralization level over time.

(A) The correlation heatmap between isotypes/subclasses and neutralization level across different SARS-CoV-2 epitopes measured by Spearman Correlation. **(B)** The correlation heatmap between OC43 -specific isotypes/subclasses and neutralization across different time courses. The adjusted p values by the Benjamini-Hochberg procedure were labeled as asterisk (* $p < 0.05$, ** $p < 0.01$, *** $p < 0.001$).

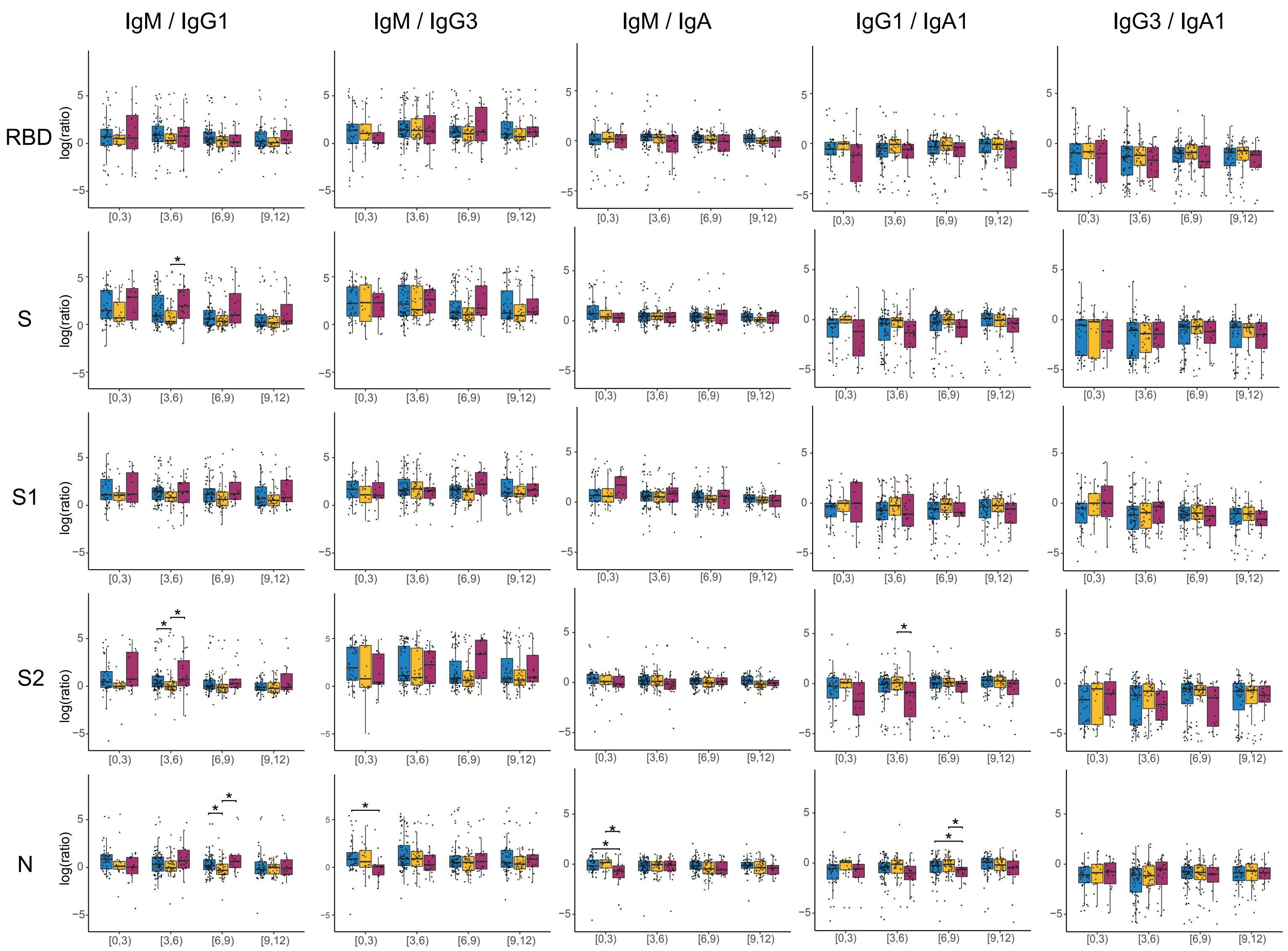

IgM / IgG1
IgM / IgG3
IgM / IgA
IgG1 / IgA1
IgG3 / IgA1
RBD
S
S1
S2
N
log(ratio)
[0,3)
[3,6)
[6,9)
[9,12)

Fig. S3. Temporal evolution of SARS-CoV-2 specific antibody.

Distributions of IgM, IgG1, IgG3, and IgA1 ratios among multiple SARS-CoV-2 specific antibodies across the different time course. A two-sample Wilcox test corrected from multiple hypothesis testing using the Benjamini-Hochberg procedure pre-time course was used to evaluate statistical differences. Significance represents the adjusted P-values: (* $p < 0.05$,** $p < 0.01$, *** $p < 0.001$).

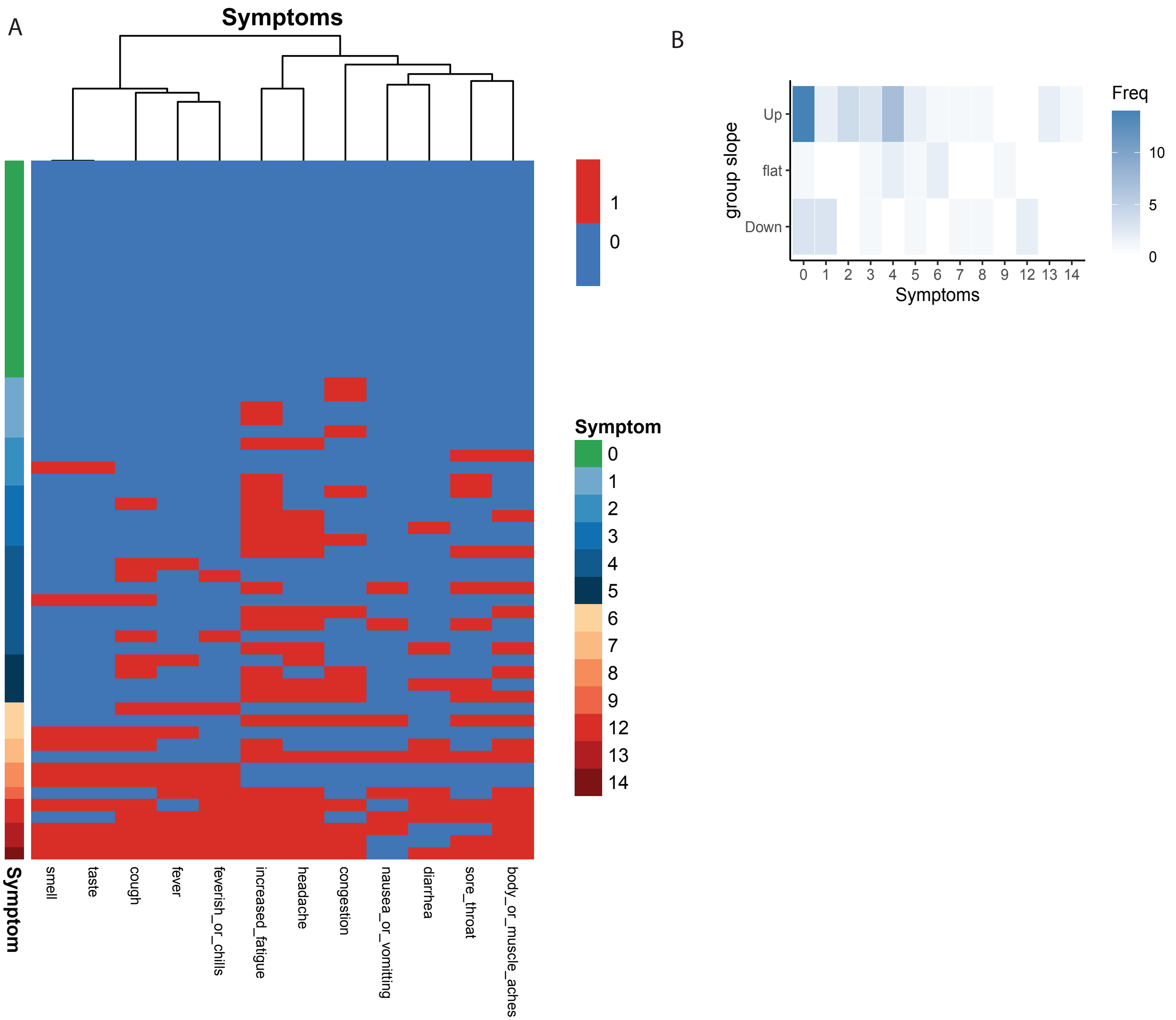
A
Symptoms
1
0
Symptom
0
1
2
3
4
5
6
7
8
9
12
13
14
smell
taste
cough
fever
feverish_or_chills
increased_fatigue
headache
congestion
nausea_or_vomitting
diarrhea
sore_throat
body_or_muscle_aches
B
Freq
10
5
0
group slope
Up
flat
Down
0 1 2 3 4 5 6 7 8 9 12 13 14
Symptoms

Fig. S4. Defined symptom groups for community acquired-COVID-19 mild and asymptomatic individuals.

The patients were divided into groups with or without COVID-19 symptoms based on 14 symptoms reported in a questionnaire. More severe COVID-19 symptoms, such as loss of smell/taste, fever, feverish/chills, or cough, were scored higher (2 points), and other, such as increased fatigue, headache, congestion, nausea/vomiting, diarrhea, sore throat, and body/muscle aches, were score with 1 point. The scale shows weighted numbers of observed symptoms and is visualized from the top (asymptomatic group: red) to bottom (an asymptomatic group with an increased number of symptoms: blue to red). **(A)** The symptom heatmap. Patients with reported clinical symptoms were marked in red, lack of symptoms was reported in blue. **(B)** The counts of relationships between defined symptoms and groups defined by the titer difference between pre-existing OC43 S-IgG1 and its corresponding titer value after SARS-CoV-2 infection.

#samples/#patients	Deceased	Severe	Moderate	Total
**Day 0-3**	14/14	21/21	43/43	78/78
**Day 3-6**	22/22	42/42	77/77	141/141
**Day 6-9**	17/17	50/50	76/76	143/143
**Day 9-12**	17/17	38/38	52/52	107/107
**Total**	70/37	151/62	248/118	469/217

Table S1. Samples distribution across disease severity and days intervals since symptoms onset.
