# Supplementary material for: Early cross-coronavirus reactive signatures of humoral immunity against COVID-19
Source: Sci Immunol. Author manuscript; Available in PMC 2022 Mar 24. (PMC8943686; doi:10.1126/sciimmunol.abj2901)
Supplement: Supplementary file 1 [file NIHMS1760475-supplement-Supplemental_Figures_S1__S2__S3__S4__table_S1__table_S2.pdf]

#samples/#patients	deceased	severe	moderate	Total
**day 0-3**	14/14	21/21	43/43	78/78
**day 3-6**	22/22	42/42	77/77	141/141
**day 6-9**	17/17	50/50	76/76	143/143
**day 9-12**	17/17	38/38	52/52	107/107
**Total**	70/37	151/62	248/118	469/217

Supplemental Table

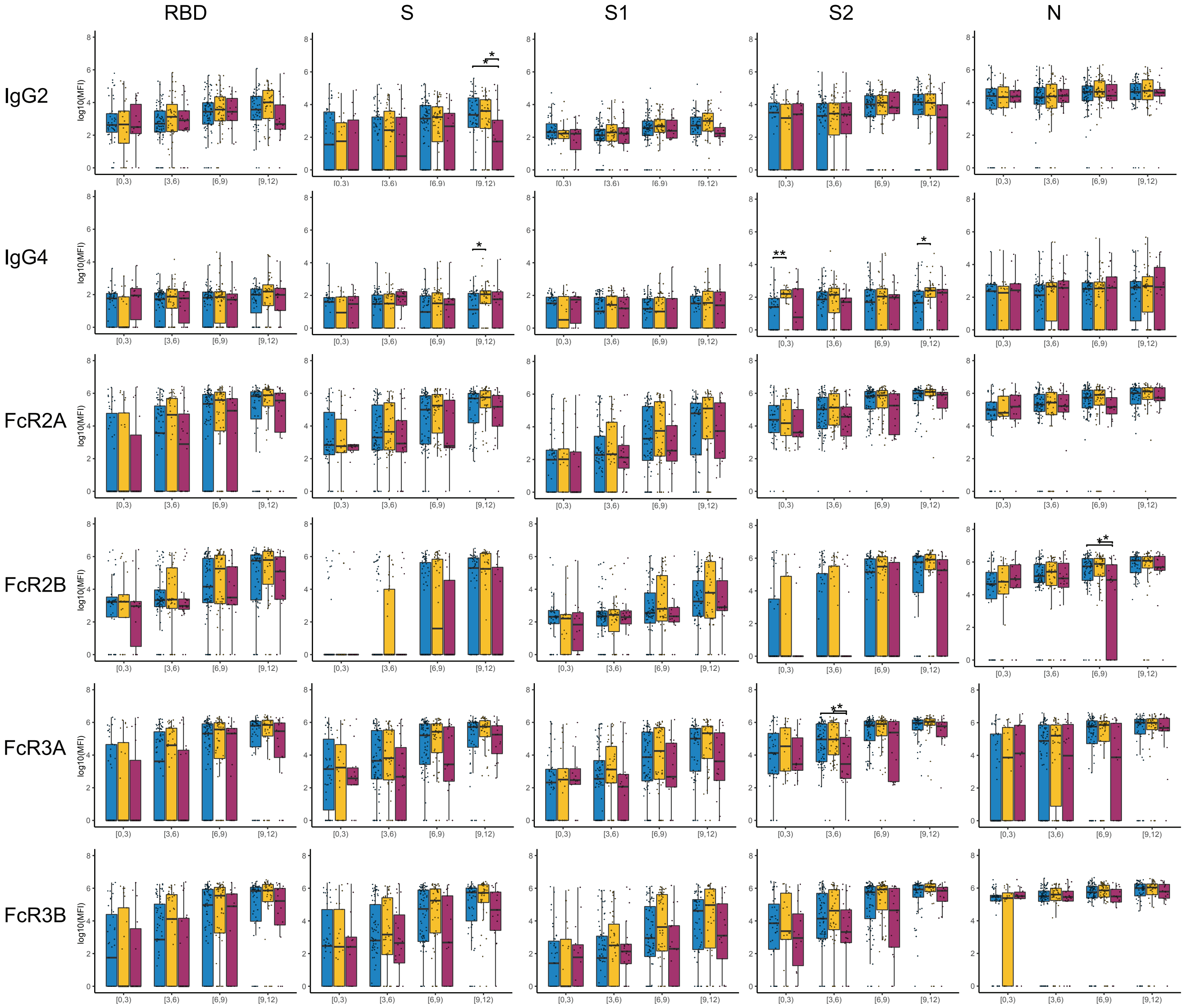

Supplemental Figure 1

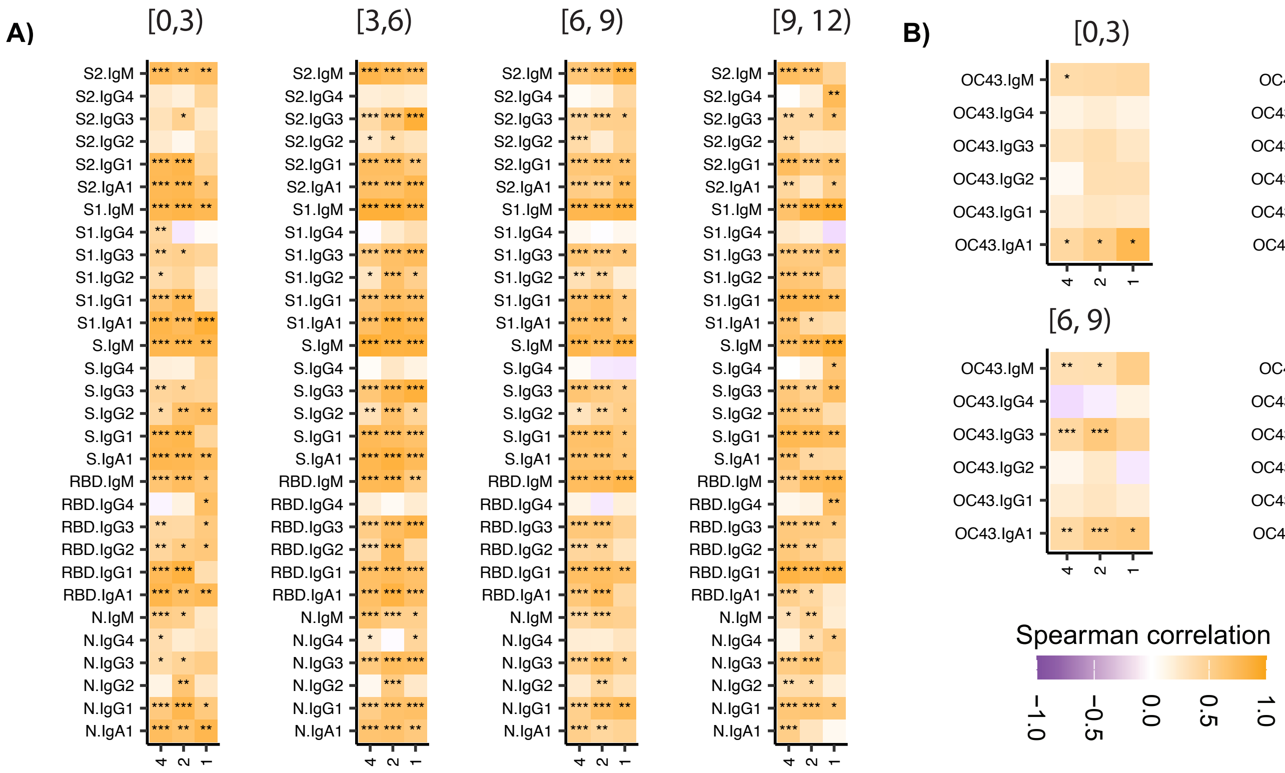

Supplemental Figure 2

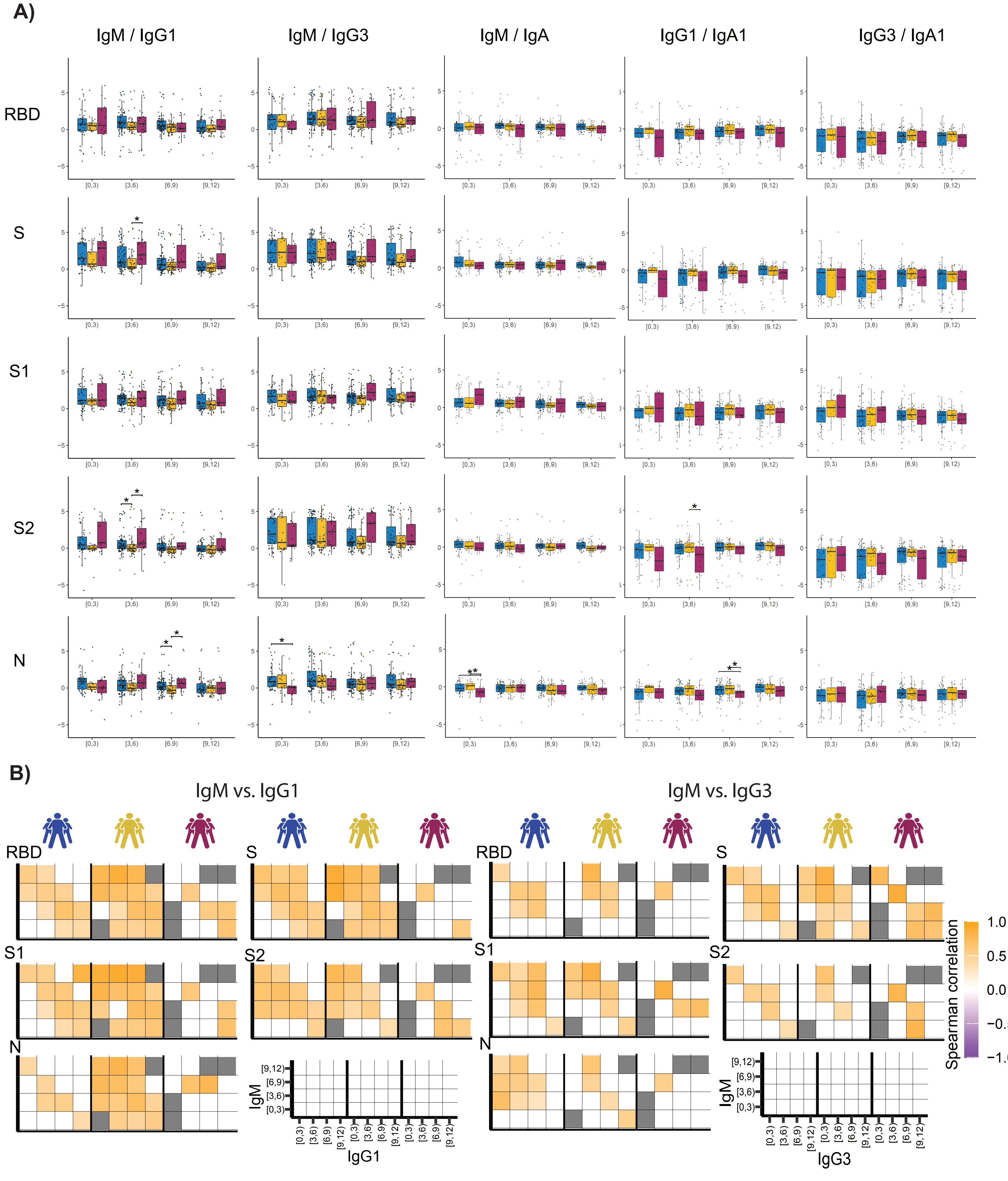

Supplemental Figure 3

(

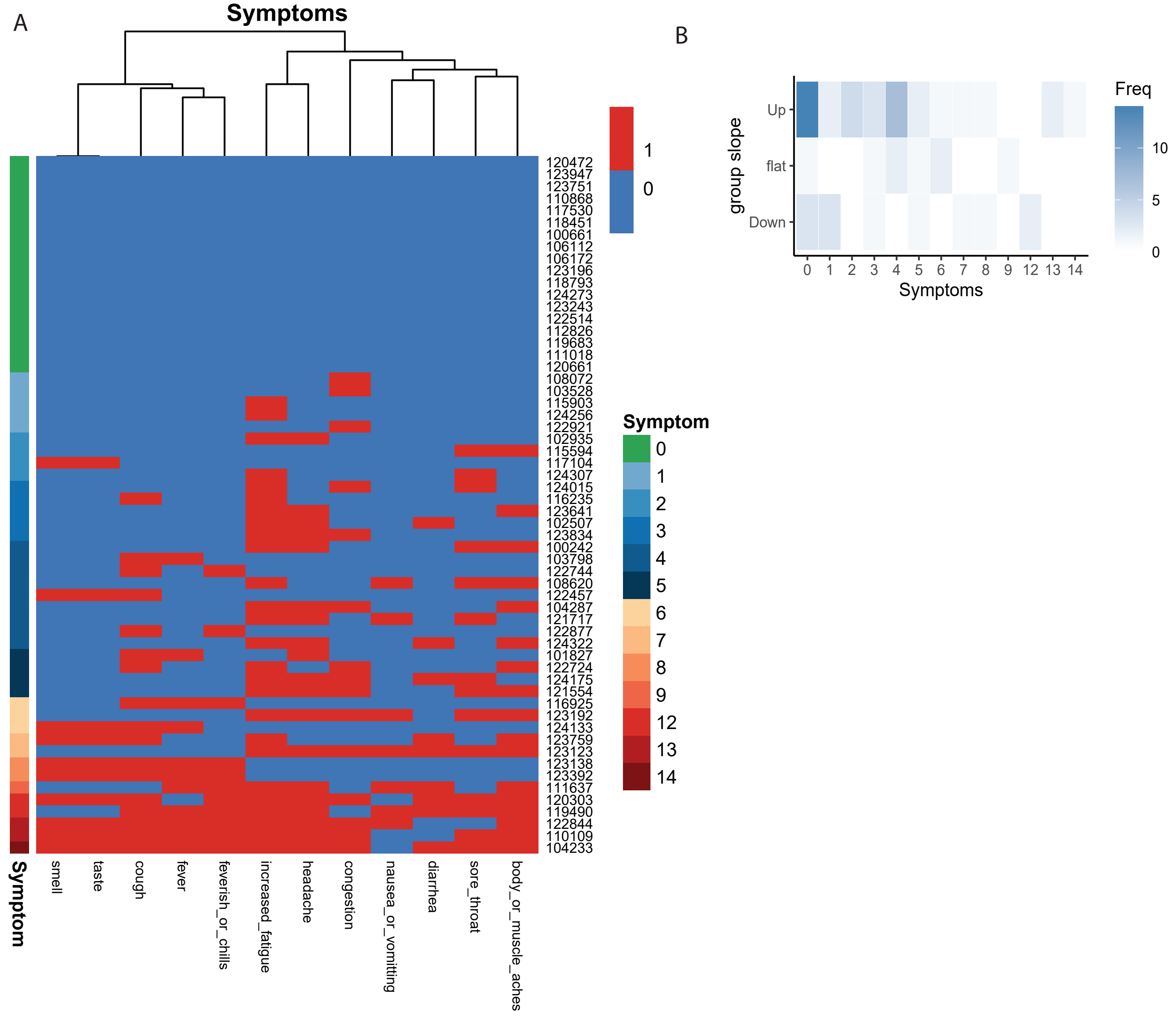

Supplemental Figure 4
